# Cellular hnRNP D promotes influenza A virus replication by inhibiting TBK1-IRF3-mediated innate immune response

**DOI:** 10.1128/jvi.00257-26

**Published:** 2026-05-13

**Authors:** Chenchen Xu, Yunling Peng, Shuhui Liu, Ran Xie, Duanchenxi Feng, Zhenwei Bi, Liping Yan

**Affiliations:** 1 MOE Joint International Research Laboratory of Animal Health and Food Safety, Institute of Immunology and College of Veterinary Medicine, Nanjing Agricultural University 70578, Nanjing, Jiangsu, China; 2 Key Laboratory of Veterinary Biological Engineering and Technology, Ministry of Agriculture and Rural Affairs, Institute of Veterinary Medicine, Jiangsu Academy of Agricultural Sciences 668638 , Nanjing, Jiangsu, China; Fred Hutchinson Cancer Center Vaccine and Infectious Disease Division, Seattle, Washington, USA

**Keywords:** influenza A virus, hnRNP D, IRF3, innate immunity, virus-host interaction

## Abstract

Heterogeneous nuclear ribonucleoproteins (hnRNPs) play important roles in the life cycle of influenza A virus (IAV). Our previous mass spectrometry analysis identified cellular hnRNP D as a novel interaction partner of the IAV polymerase basic 2 (PB2) protein. However, the functional implications of hnRNP D in IAV replication and the underlying mechanisms remained unknown. In this study, we confirmed that hnRNP D directly interacts with the PB2 protein of the A/Puerto Rico/8/1934 (PR8, H1N1) strain, while also binding to other proteins within viral ribonucleoprotein complexes (vRNPs). These interactions collectively inhibit vRNPs assembly and viral polymerase activity. However, we have found that hnRNP D enhances the viral titer of IAV in A549 cells. Mechanistically, hnRNP D suppresses the activation of the interferon (IFN)-β promoter, and the mRNA levels of downstream factors in the type I IFN signaling pathway. In detail, hnRNP D inhibits IFN-β promoter activation induced by crucial antiviral proteins and interacts strongly with the interferon regulatory factor 3 (IRF3). More importantly, hnRNP D blocks the binding of TANK-binding kinase 1 (TBK1) to IRF3, thereby impeding the phosphorylation and activation of IRF3. Collectively, we unveil a novel viral immune evasion strategy by which IAV hijacks a host RNA-binding protein, hnRNP D, to facilitate self-replication. This work not only elucidates the intricate trade-off mechanisms in virus-host interaction but also identifies hnRNP D as a potential therapeutic target aimed at bolstering antiviral immunity.

**IMPORTANCE:**

Influenza viruses are serious zoonotic pathogens, causing millions of severe infections and hundreds of thousands of deaths annually. The hnRNP family is known to influence IAV replication and pathogenesis. Here, we demonstrate for the first time that hnRNP D functions as a positive factor for IAV replication. We show that hnRNP D exerts a net promoting effect on viral replication primarily by suppressing the type I interferon response. The mechanism of action involves viral infection upregulating hnRNP D, which interacts with the key transcription factor IRF3, disrupting the TBK1-IRF3 signaling axis. Our findings provide novel insights into how a host RNA-binding protein can be co-opted by IAV to dampen antiviral innate immunity, presenting a potential target for developing new therapeutic strategies against influenza.

## INTRODUCTION

Influenza A viruses (IAVs) are highly contagious respiratory pathogens, which have caused several worldwide pandemics, such as the H1N1 “Spanish flu” of 1918 and the H1N1 pandemic of 2009 (1–4). Nearly 10% of the world’s population is affected by influenza annually, leading to millions of severe cases and hundreds of thousands of deaths and causing serious public health concerns (5).

The IAV genome consists of eight single-stranded, negative-sense RNA segments. These fragments encode multiple proteins, including the three subunits of the viral RNA-dependent RNA polymerase (RdRp): polymerase basic 1 (PB1), polymerase basic 2 (PB2), and polymerase acidic (PA), along with the nucleoprotein (NP) (6, 7). Together, the polymerase complex with NP and viral RNA (vRNA) forms viral ribonucleoprotein complexes (vRNPs) that are essential for viral transcription and replication (8). The PA subunit possesses endonuclease activity critical for “cap-snatching,” PB1 contains the catalytic center for RNA synthesis, and PB2 is responsible for binding capped primers and determining the host range of IAV (9–11). Importantly, the interaction of PB2 with numerous host cellular factors throughout the viral life cycle underscores its central role in virus-host interactions, offering a promising avenue for developing novel antiviral strategies (12–14).

Heterogeneous nuclear ribonucleoproteins (hnRNPs) constitute a large and functionally diverse family of RNA-binding proteins (RBPs), playing crucial roles in multiple stages of RNA metabolism (15). Comprising more than 20 members (designated hnRNP A1 to U), with molecular weights ranging from 34 kD to 120 kD, this family has been increasingly implicated for its role in the replication cycles of various viruses, including IAV (16–21). It is noteworthy that different hnRNP proteins exert unique and sometimes contradictory effects on the IAV life cycle. For instance, hnRNP AB is believed to restrict viral replication by blocking the nuclear export of IAV mRNA (22, 23), while hnRNP A2/B1, despite its ability to interact with the NP protein and enhance polymerase activity, ultimately inhibits the replication of IAV (24, 25). In contrast, other family members promote viral replication. HnRNP M enhances efficient transcription of specific influenza viral gene segments in A549 cells (26), and hnRNP K promotes viral mRNA splicing and M2 expression, thereby positively regulating viral replication (21). HnRNP D (also known as AUF1) is a key regulator of mRNA splicing and stability, thereby regulating gene expression at the transcriptional and translational levels (27, 28). Our previous study has identified hnRNP D, along with hnRNP A2/B1 and hnRNP AB, as interactors of the IAV PB2 protein (29). While the effects of hnRNP A2/B1 and hnRNP AB on IAV replication have been previously characterized (22, 23, 25), the specific function of hnRNP D in IAV life cycle and the mechanistic basis for its potential role remain entirely unknown.

The host innate immune system serves as the first line of defense against viral invasion. Viral RNA or DNA in infected cells is recognized by a variety of pattern recognition receptors (PRRs), including toll-like receptors (TLRs), nucleotide-binding oligomerization domain (NOD)-like receptors (NLRs), and retinoic acid-inducible gene I (RIG-I)-like receptors (RLRs), which activate downstream signaling responses and ultimately induce the secretion of cytokines such as type I interferon (IFN) and inflammatory factors (30, 31). These intracellular signaling cascades render cells into an antiviral state. RIG-I plays a crucial function in recognizing IAV RNAs and inducing antiviral cytokine secretion in epithelial cells (32, 33). Upon recognizing IAV RNA, RIG-I undergoes conformational changes, subsequently recruiting the mitochondrial antiviral signaling protein (MAVS). Activated MAVS further binds to TNF receptor associated factor 3 (TRAF3), leading to the phosphorylation and nuclear translocation of interferon regulatory factor 3 (IRF3) via inhibitor of nuclear factor kappa-B kinase ε (IKKε)/TANK-binding kinase 1 (TBK1). Phosphorylated IRF3 dimerizes, translocates to the nucleus, and drives the expression of type I interferons (IFNs) and interferon-stimulated genes (ISGs), establishing an antiviral state (33–37). While immunoregulatory potential of certain hnRNP proteins has been reviewed, the possibility of targeting hnRNP D to modulate antiviral innate immunity has not been explored (38). This represents a critical gap in our understanding of how RNA-binding proteins fine-tune the host response to influenza infection.

To address these gaps, this study aimed to systematically investigate the role of hnRNP D in IAV infection. We identified hnRNP D as a novel interactor of the IAV vRNP complex and discovered its unexpected dual functionality: it directly inhibits viral polymerase activity while simultaneously promoting viral replication by potently suppressing the IRF3-mediated type I interferon response. Therefore, this study reveals a novel mechanism by which IAV utilizes hnRNP D for immune evasion to facilitate its own replication, providing potential targets for antiviral immune responses while also identifying new host functions involved in IAV replication.

## RESULTS

### Host protein hnRNP D interacts with PB2 of IAV

**Fig 1:**
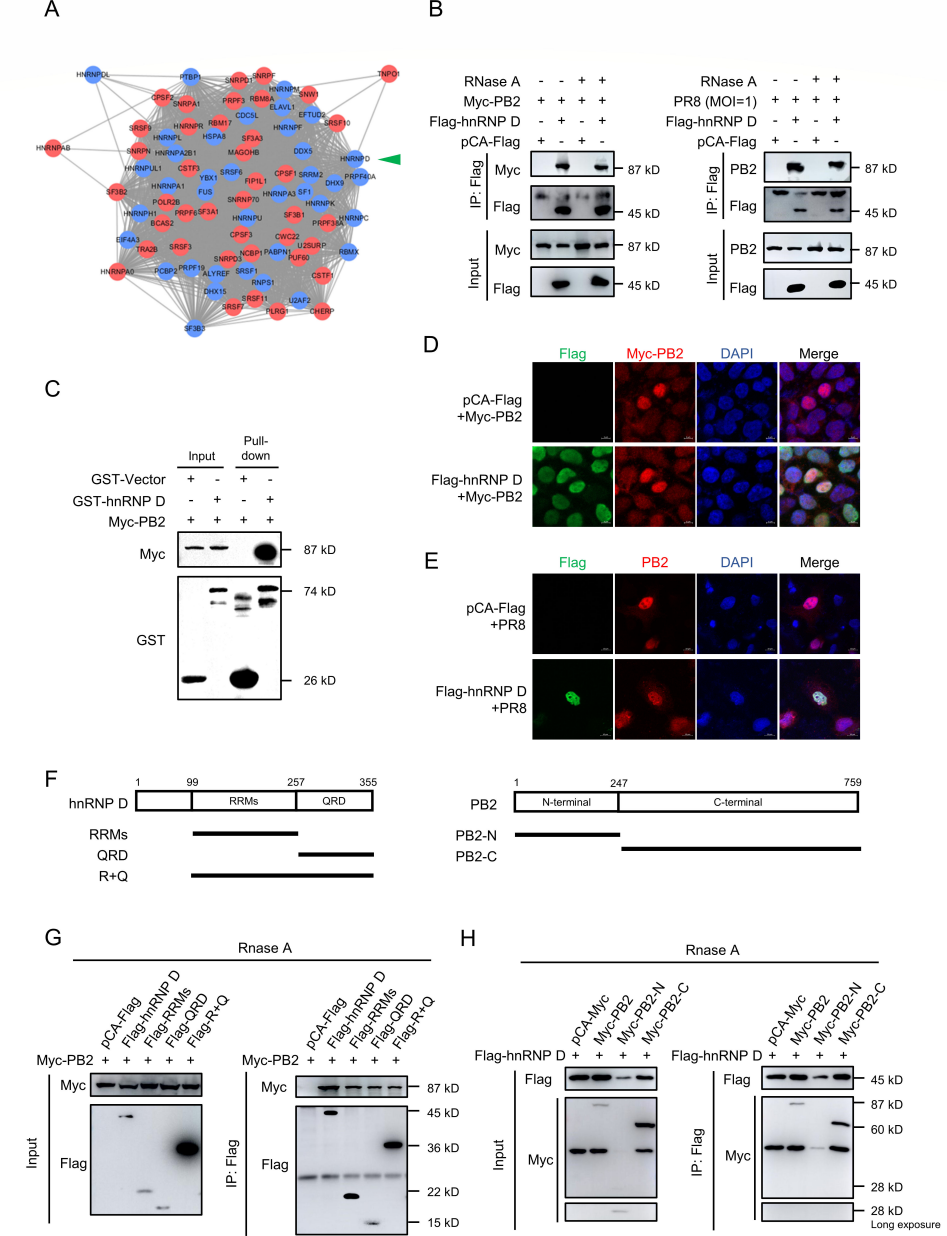
The interaction between hnRNP D and IAV PB2 protein. (**A**) The host proteins interaction network with PR8 PB2. PR8 PB2 protein-host protein interaction map generated using Cytoscape software. Red nodes indicate host proteins interacting with PB2 protein in the presence of human ANP32A. Blue nodes indicate host proteins that interact with the PB2 protein regardless of whether human or avian ANP32A is present. (**B**) HnRNP D interacted with exogenous and endogenous PB2. HEK293T cells were co-transfected with Flag-hnRNP D and Myc-PB2 for 24 h (Left), and A549 cells were transfected with flag-hnRNP D for 24 h and then inoculated with PR8 (MOI = 1) for 8 h (Right). The cell lysates were treated with or without RNase A prior to IP with an anti-Flag antibody. (**C**) Direct interaction shown by GST pull-down. After HEK293T transfection of Myc-PB2 for 36 h, cell lysates were co-incubated with prokaryotic expression of GST-hnRNP D for GST pull-down. (**D and E**) Co-localization of hnRNP D and PB2. (**D**) HEK293T cells were co-expressing Flag-hnRNP D and Myc-PB2 for 24 h, and the co-localization of Flag-hnRNP D (green) and Myc-PB2 (red) was detected by confocal laser scanning. DAPI, blue. Scale bar, 5 μm. (**E**) A549 cells were transfected with flag-hnRNP D for 24 h, inoculated with PR8 (MOI = 1) for 8 h, and then conducted immunofluorescence staining and confocal microscopy. Flag-hnRNP D, green. PB2, red. DAPI, blue. Scale bar, 10 μm. (**F**) Schematic of hnRNP D truncations (RRMs, QRD, R + Q) and PB2 truncations (N-terminal, C-terminal). (**G**) Interaction of hnRNP D truncations with PB2. Flag-hnRNP D, Flag-RRMs, Flag-QRD, Flag-R+Q or pCA-Flag were co-transfected with Myc-PB2 in HEK293T cells for 24 h, and the whole cell lysate was treated with RNase A. The interaction was identified by co-immunoprecipitation using anti-Flag antibody. (**H**) Interaction of PB2 truncations with hnRNP D. Myc-PB2, Myc-PB2-N, Myc-PB2-C, or pCA-Myc were co-transfected with Flag-hnRNP D in HEK293T for 24 h, and the whole cell lysate was treated same as for panel G. The data displayed are representative of three independent experiments.

In our mass spectrometry screening of host proteins interacting with IAV PB2 protein, hnRNP D was identified as a novel candidate binding protein, an association previously unreported in influenza virus research (Fig. 1A). HnRNP D consists of four isoforms (p37, p40, p42, and p45) generated by alternative pre-mRNA splicing (Fig. S1) and is widely distributed throughout human tissues (39). Sequencing results confirmed that the predominant variant amplified from A549 cells was the p45 subtype (NCBI Reference Sequence: NM_031370.3). Based on this sequence, we constructed an expression plasmid of hnRNP D. Co-immunoprecipitation (Co-IP) assays in mammalian cells verified that hnRNP D interacted with both exogenous and endogenous PB2 protein (Fig. 1B). This interaction was further validated by using GST pull-down assay (Fig. 1C) and was shown to be independent of RNA bridging, as treatment with RNase A did not disrupt the complex (Fig. 1B). Immunofluorescence microscopy demonstrated clear co-localization of hnRNP D with PB2 in the cellular context (Fig. 1D and E). HnRNP D contains two RNA recognition motifs (RRMs) and a Glutamine-rich domain (QRD). To identify the interacting domains, we constructed truncation mutants of hnRNP D (RRMs, QRD, R + Q) (Fig. 1F). Co-IP results indicated that all hnRNP D truncations retained the ability to bind PB2 in an RNA-independent manner (Fig. 1G; Fig. S2A). We also identified the specific motif of PB2 interacting with hnRNP D, and the C-terminus of PB2 was revealed to be the interaction domain (Fig. 1H; Fig. S2B). Collectively, the above data demonstrated hnRNP D as a novel functional partner of the IAV PB2 protein.

### HnRNP D inhibits the polymerase activity and vRNP assembly of PR8 strain

**Fig 2:**
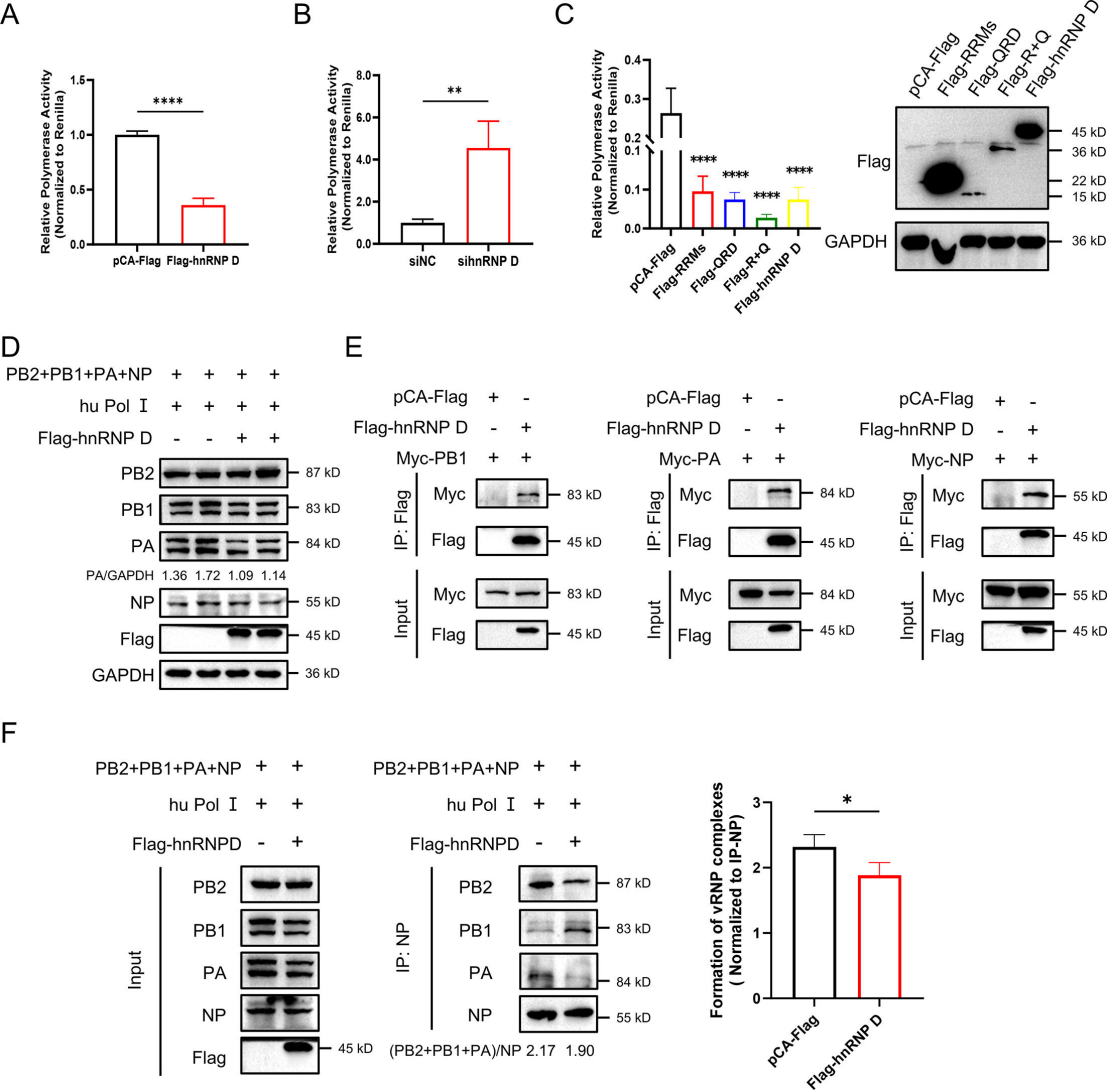
HnRNP D inhibits viral polymerase activity and vRNP assembly. (**A–C**) The effect of hnRNP D on PR8 polymerase activity. HEK293T cells were co-transfected with polymerase genes, phuPol I-Luc, RL-TK, and Flag-hnRNP D (**A**) or sihnRNP D (**B**) or hnRNP D mutants (**C**) for 24 h, the polymerase activity was detected by Dual Luciferase Reporter Gene Assay. The expression of truncated hnRNP D mutants was detected by Western blotting. (**D**) The effects of hnRNP D on protein levels of viral polymerase components and NP. HEK293T cells were co-transfected with the polymerase genes, phuPol I-Luc, and Flag-hnRNP D plasmids. After 24 h, cells were lysed to detect levels of PB2, PB1, PA, and NP proteins. (**E**) HnRNP D interacted with PB1, PA, and NP. The Myc-tagged PB1, PA, or NP were co-transfected with Flag-hnRNP D into HEK293T cells, and 24 h later, cells were lysed for Co-IP assay with anti-Flag antibody. (**F**) HnRNP D disrupted vRNP assembly. The vRNP genes, phuPol I-Luc, and Flag-hnRNP D plasmids were co-transfected into HEK293T cells for 24 h. Co-IP was performed using anti-NP antibody to detect the protein levels of PB2, PB1, and PA. The levels of co-precipitated PB2, PB1, and PA were quantified using Image J software. The data displayed are representative of three independent experiments. Bars represented the mean ± SD (*n* = 3). Values were analyzed by Student’s *t*-test (**A, B, F**) and one-way ANOVA (**C**). *P < 0.05, **P < 0.01, ****P < 0.0001.

Given the interaction between hnRNP D and PB2, a crucial component of the IAV RdRp, we next explored its functional impact. The polymerase activity assay revealed that overexpressing hnRNP D significantly suppressed the polymerase activity of PR8 strain (Fig. 2A). Consistent with this, siRNA-mediated knockdown of endogenous hnRNP D enhanced viral polymerase activity (Fig. 2B). We subsequently transfected truncated variants of hnRNP D into HEK293T cells to identify the functional domains involved. The outcomes demonstrated that both the RRMs and QRD contributed to this suppression (Fig. 2C). The examination of viral protein expression showed that the overexpression of hnRNP D did not alter the levels of PB2, PB1, and NP but specifically reduced PA accumulation (Fig. 2D). The interactions between hnRNP D and the other three proteins except PB2 were further verified in HEK293T cells. Co-IP assays demonstrated expanded the interaction network, revealing that hnRNP D interacted with all viral proteins within vRNP (Fig. 1B and 2E), but only directly interacted with PB2 (Fig. 1C; Fig. S3). Based on these multiple interactions, we hypothesized that hnRNP D might influence the assembly of vRNP. So, we performed Co-IP assay using NP antibody to precipitate PA, PB1, and PB2. As expected, hnRNP D overexpression significantly reduced the co-precipitation of PA, PB1, and PB2 with NP (Fig. 2F), indicating that hnRNP D impaired the assembly of the vRNP complex. Taken together, these results demonstrated that hnRNP D had the function of suppressing polymerase activity and vRNP assembly of IAV.

### HnRNP D facilitates IAV replication in A549 cells

**Fig 3:**
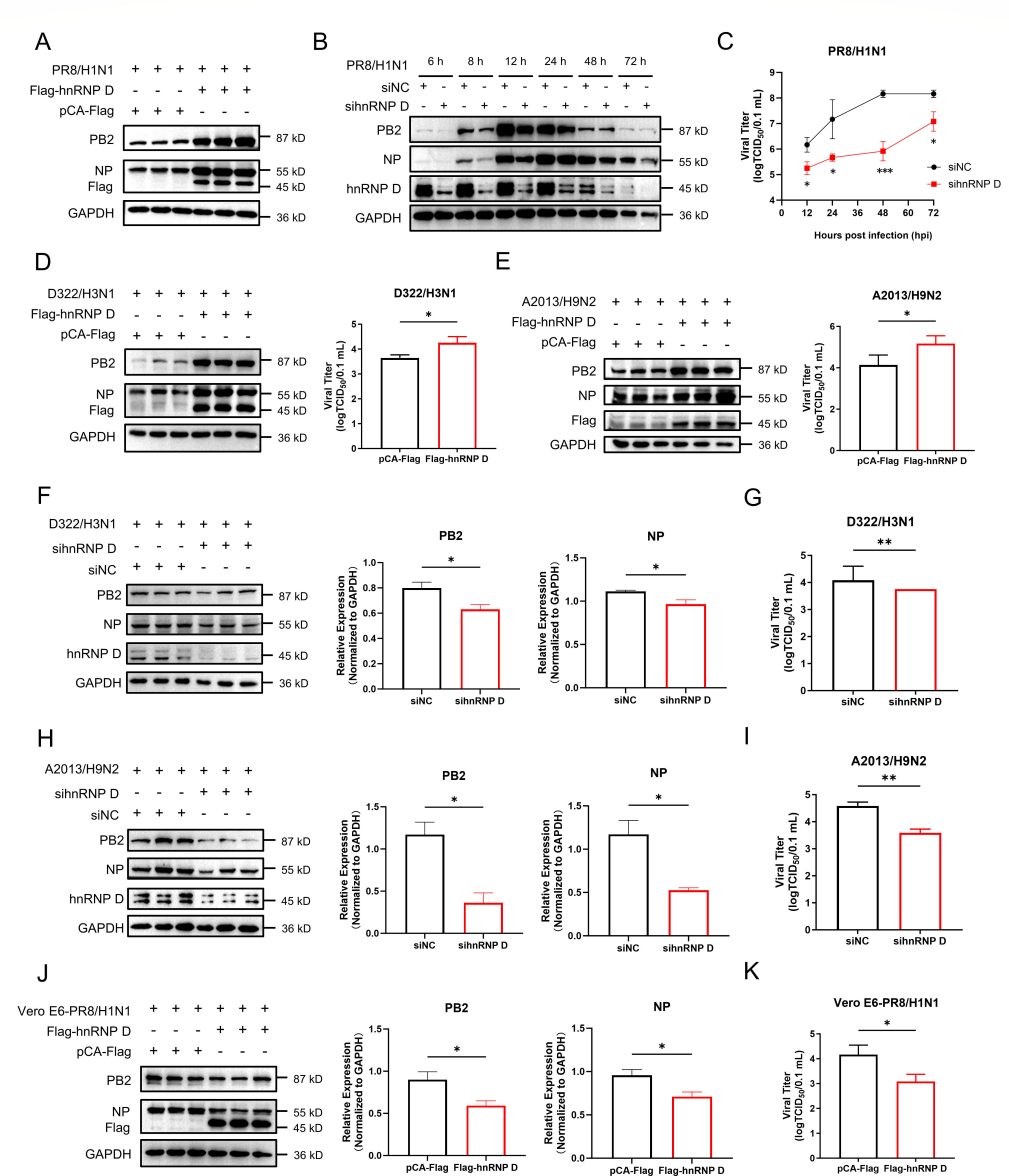
HnRNP D promotes the replication of multiple IAV subtypes in A549 cells. (**A–C**) The effects of hnRNP D on PR8 replication. (**A**) Overexpression of hnRNP D increased viral PB2 and NP protein levels. A549 cells were overexpressed Flag-hnRNP D for 36 h and then infected with PR8 (MOI = 0.01) for 12 h. The PB2 and NP protein levels were detected by Western blotting. (**B and C**) Knockdown of hnRNP D inhibited PR8 replication. Endogenous hnRNP D in A549 cells was disrupted using sihnRNP D for 36 h, followed by inoculation with PR8 (MOI = 0.01) for 12 h. The PB2 and NP protein levels were detected by Western blotting (**B**), and the viral titer of supernatant were determined in MDCK cells by Reed-Muench method (**C**). (**D and E**) Overexpression of hnRNP D promoted the replication of other subtypes of IAV. A549 cells were over transfected Flag-hnRNP D for 36 h, followed by inoculation with D322 (**D**) or A2013 (**E**) for 12 h. The PB2 and NP protein levels were detected by Western blotting, and the viral titer of supernatant were determined in MDCK cells by Reed-Muench method. (**F–I**) Knockdown of hnRNP D inhibited the replication other subtypes of IAV. Thirty-six hours after transfection of A549 cells with sihnRNP D, cells were inoculated with D322 (**F and G**) or A2013 (**H and I**) for 12 h (MOI = 0.01). The PB2 and NP expression (**F and H**) was detected by Western blotting and was analyzed using Image J software. The viral titer (**G and I**) of supernatant were determined in MDCK cells by Reed-Muench method. (**J and K**) Overexpression of hnRNP D suppressed the replication of PR8 strain in Vero E6 cells. Flag-hnRNP D was transfected into Vero E6 cells for 36 h, followed by inoculation with PR8 at MOI = 0.01 for 12 h. The expression of relevant proteins (**J**) was detected by Western blotting and was analyzed using Image J software. The viral titer (**K**) of supernatant were determined in MDCK cells by Reed-Muench method. The data displayed are representative of three independent experiments. Bars represented the mean ± SD (*n* = 3). Values were analyzed by Student’s *t*-test. *P < 0.05, **P < 0.01, ***P < 0.001.

Based on the inhibitory effect of hnRNP D on the viral polymerase, we next investigated its effects on the complete viral replication cycle. Unexpectedly, overexpression of hnRNP D in A549 cells resulted in a significant increase in the levels of viral PB2 and NP protein following PR8 infection (Fig. 3A). This promoting effect was reversed when siRNA was used to knock down endogenous hnRNP D expression (Fig. 3B), and hnRNP D knockdown significantly decreased viral titers in the supernatant (Fig. 3C). To verify the generalized effect of this phenomenon, we screened two other subtypes of IAVs, including A/Anser fabalis/China/D322/2020 (D322, H3N1) and A/chicken/Zhejiang/A2013/2017 (A2013, H9N2). In both cases, overexpression of hnRNP D enhanced viral protein synthesis and progeny virus production (Fig. 3D and E), while its knockdown had the opposite effect (Fig. 3F through I). These consistent findings across multiple viral strains confirmed hnRNP D as a promoter that facilitated IAV replication in A549 cells, creating a paradox with its direct inhibitory effect on the polymerase complex. Host proteins can exert dual regulatory mechanisms against IAV by modulating innate immunity and other signaling pathways (40). To resolve the contradiction between hnRNP D’s suppression of polymerase activity and its overall proviral phenotype, we hypothesized that its proviral phenotypes in A549 cells might be associated with innate immune responses. In Vero E6 cells (an interferon secretion-deficient cell line), we found that overexpression of hnRNP D reduced the expression levels of PB2, NP proteins, and viral titers of PR8 strain (Fig. 3J and K). The above results indicated that in the absence of interferon, hnRNP D could resist IAV infection by inhibiting polymerase activity; whereas in typical lung cells, hnRNP D promoted IAV replication in an interferon-dependent manner.

### HnRNP D inhibits the production of IFN-β in innate immune signaling pathways

**Fig 4:**
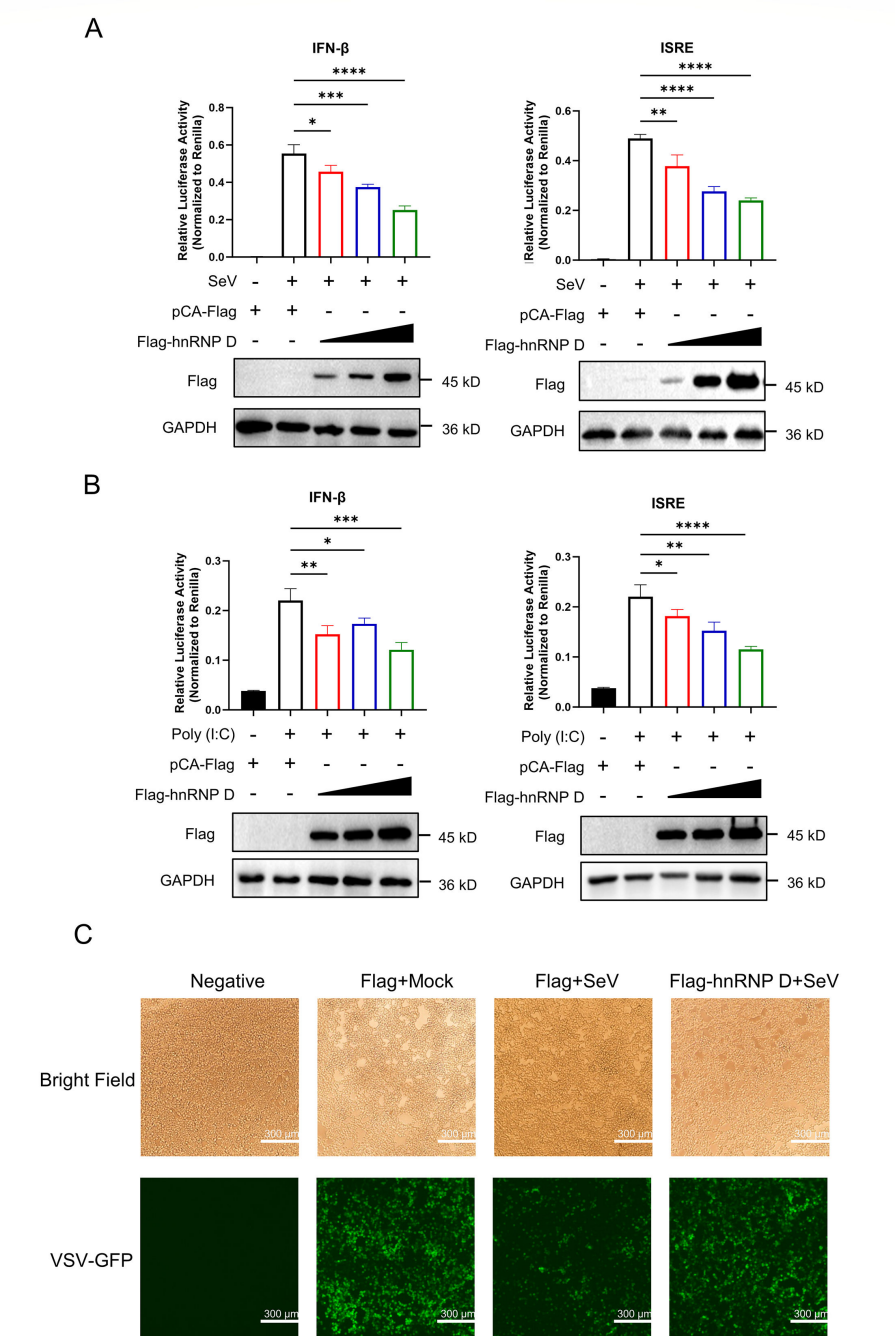
HnRNP D suppresses type I IFN signaling pathway. (**A and B**) Overexpression of hnRNP D suppressed the activation of IFN-β promoter and ISRE triggered by SeV (**A**) and Poly (I:C) (**B**). HEK293T cells were co-transfected with IFN-β promoter or ISRE luciferase reporter, RL-TK and a graded dose of Flag-hnRNP D. After 24 h transfection, cells were infected with SeV (MOI = 0.1, **A**) or transfected with Poly (I:C) (3 μg, **B**) for 12 h. The cells lysates were identified by dual luciferase reporter gene assay. The expression levels of relevant proteins were detected using Western blotting analysis. (**C**) Overexpression of hnRNP D promoted the replication of VSV-GFP. HEK293T cells were infected with SeV (MOI = 0.1) for 12 h after overexpressing Flag-hnRNP D for 24 h. UV irradiated cell supernatants were inoculated with fresh cells for 24 h. The cells then were inoculated with VSV-GFP (MOI = 0.01) for 24 h and observed under fluorescence microscope. Scale bar, 300 μm. The data displayed are representative of three independent experiments. Bars represented the mean ± SD (*n* = 3). Values were analyzed by one-way ANOVA. *P < 0.05, **P < 0.01, ***P < 0.001, ****P < 0.0001.

**Fig 5:**
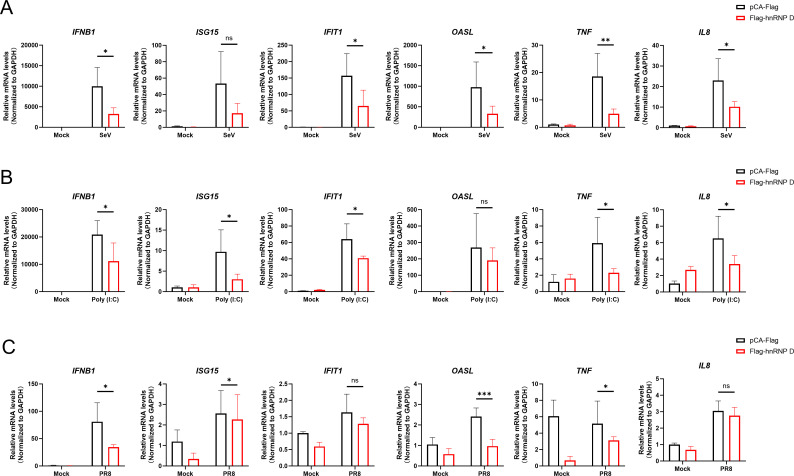
HnRNP D represses the mRNA levels of ISGs and inflammatory cytokines. A549 cells were transfected with flag-hnRNP D for 36 h and subsequently stimulated with SeV (MOI = 0.1, **A**), Poly (I:C) (3 μg, **B**), or PR8 (MOI = 0.1, **C**). Cell RNA was extracted and subjected to quantitative real-time PCR. The data displayed are representative of three independent experiments. Bars represented the mean ± SD (*n* = 5). Values were analyzed by Student’s *t*-test. Ns represented no significance, *P < 0.05, **P < 0.01, ***P < 0.001, ****P < 0.0001.

Next, we investigated the potential role of hnRNP D in regulating the host antiviral response. Overexpression of hnRNP D dose-dependently suppressed the activation of IFN-β promoter and interferon-stimulated response element (ISRE) promoter triggered by Sendai virus (SeV, Fig. 4A) and Poly (I:C) (Fig. 4B), whereas interference with hnRNP D promoted both response (Fig. S4A and B). Observation of GFP signals revealed that following overexpression of hnRNP D in HEK293T cells, cell supernatants inactivated by UV irradiation promoted the replication of the GFP-expressing vesicular stomatitis virus (VSV-GFP), whereas this effect was absent upon interference with hnRNP D (Fig. 4C; Fig. S4C). This indicated that hnRNP D possessed the ability to reduce the secretion of antiviral factors. Consistent with this, quantitative PCR analysis showed that overexpression of hnRNP D significantly repressed the transcription of interferon beta 1 (IFNB1), interferon-stimulated gene 15 (ISG15), interferon-induced protein with tetratricopeptide repeats 1 (IFIT1), oligoadenylate synthase-like protein (OASL), tumor necrosis factor (TNF), and interleukin8 (IL8) genes which triggered by SeV, Poly (I:C), and PR8 in A549 cells (Fig. 5A through C). Conversely, knocking down hnRNP D produced the opposite effect, enhancing the expression of these immune genes (Fig. S5A through C). Taken together, these data unequivocally indicated that the host protein hnRNP D acted as a broad negative regulator of the type I IFN immune responses activated by RNA viruses.

### HnRNP D attenuates the innate immune response by targeting IRF3 phosphorylation

**Fig 6:**
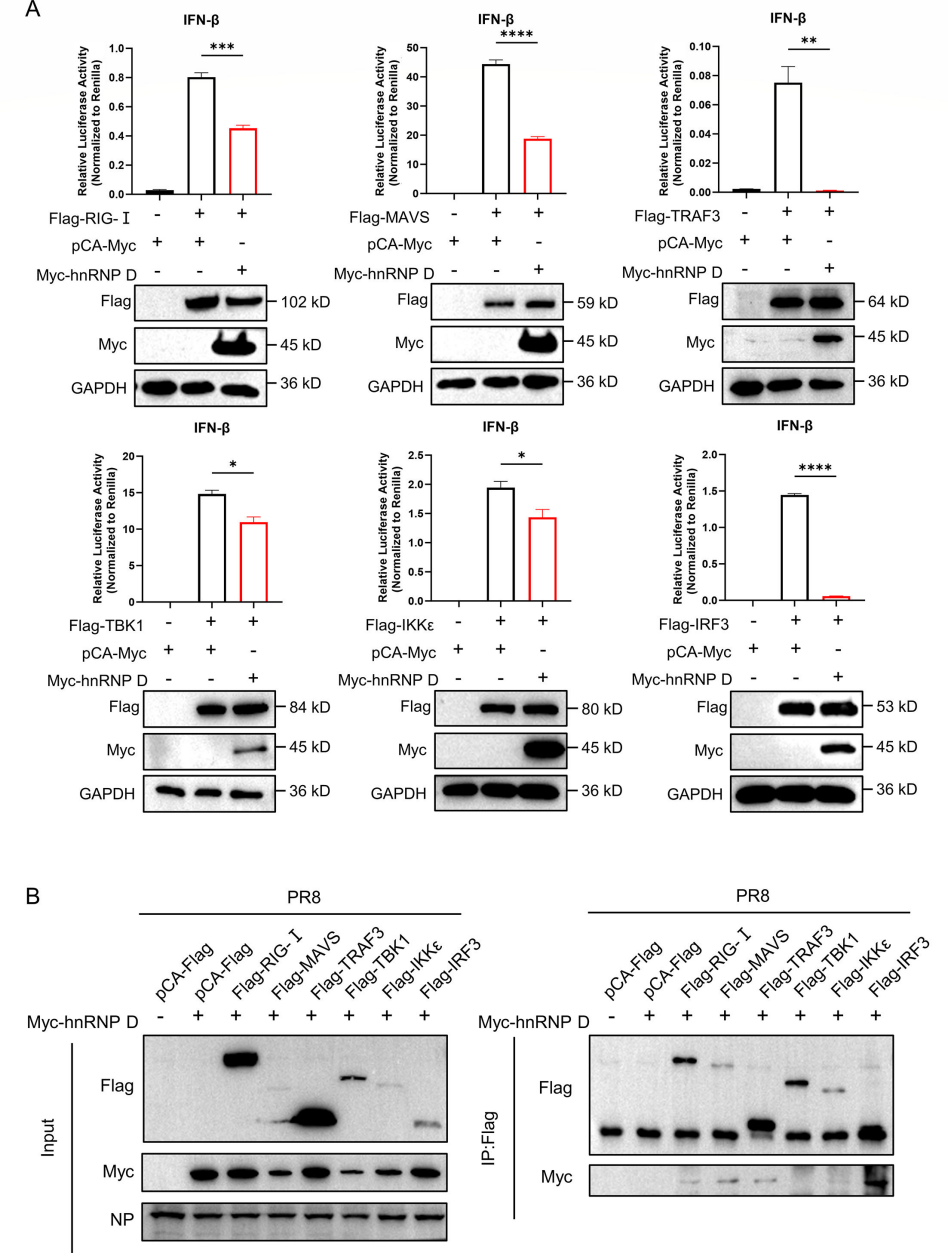
HnRNP D inhibits activation of the RIG-I-IRF3-IFN-β axis. (**A**) Overexpression of hnRNP D repressed RIG-I-to-IRF3-mediated activation of the IFN-β promoter. Flag-tagged RIG-I, MAVS, TRAF3, TBK1, IKKε, or IRF3, along with Myc-hnRNP D, IFN-β-Luc, and RL-TK, were co-transfected into HEK293T cells. After 24 h, cells were lysed and subjected to dual luciferase reporter gene assay. The expression levels of relevant proteins were detected using Western blotting analysis. (**B**) HnRNP D interacted with key proteins in the RIG-1-IRF3 axis. HEK293T cells were co-transfected with Flag-tagged RIG-I, MAVS, TRAF3, TBK1, IKKε, or IRF3, and Myc-tagged hnRNP D for 24 h, followed by infection with PR8 (MOI = 0.01) for 12 h. Co-IP was performed using anti-Flag antibody, and Western blotting analysis was conducted to detect the expression of relevant proteins. The data displayed are representative of three independent experiments. Bars represented the mean ± SD (*n* = 3). Values were analyzed by Student’s *t*-test. *P < 0.05, **P < 0.01, ***P < 0.001, ****P < 0.0001.

**Fig 7:**
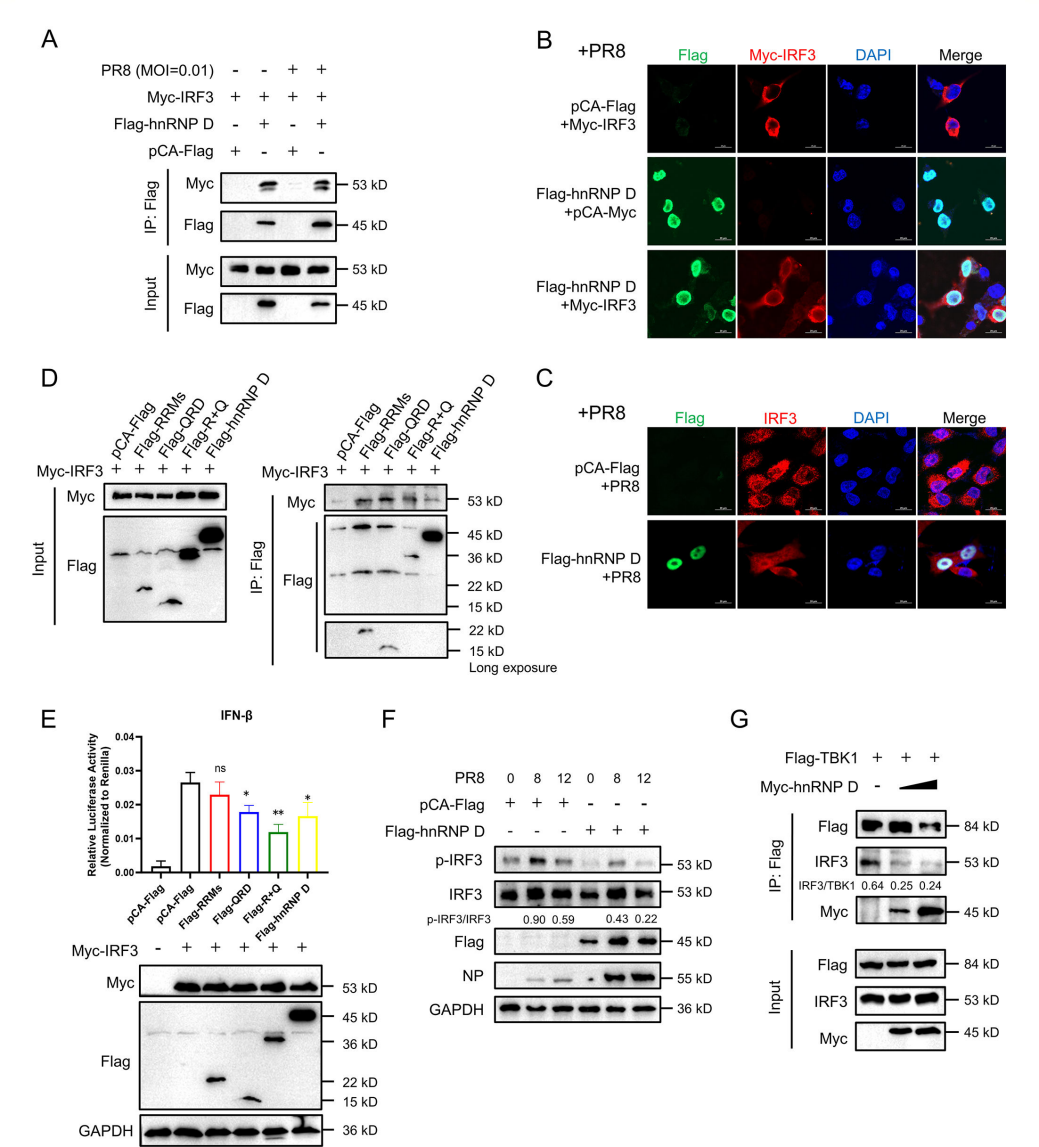
HnRNP D blocks TBK1-IRF3 interaction to inhibit IRF3 phosphorylation. (**A**) HnRNP D interacted with IRF3. Flag-hnRNP D and Myc-IRF3 were co-transfected into HEK293T cells. After 24 h, cells were infected or uninfected with PR8 (MOI = 0.01) for 12 h and then were lysed and immunoprecipitated using anti-Flag antibody. Co-IP protein expression was detected by Western blotting. (**B and C**) Identification of subcellular localization of hnRNP D and IRF3. (**B**) Co-transfection of Flag-hnRNP D and myc-IRF3 in HEK293T cells, followed by PR8 (MOI = 1) infection 8 h after 24 h transfection. The subcellular localization of hnRNP D and IRF3 was detected by confocal laser scanning. Flag-hnRNP D, green. Myc-IRF3, red. DAPI, blue. Scale bar, 20 μm. (**C**) Flag-hnRNPD was transfected into A549 cells for 36 h, followed by infection with PR8 (MOI = 1) for 8 h. Protein localization was detected using confocal laser scanning. Flag-hnRNP D, green. IRF3, red. DAPI, blue. Scale bar, 20 μm. (**D**) Identification of the domain where hnRNP D interacted with IRF3. After co-transfecting Flag-tagged truncated hnRNP D and Myc-IRF3 into HEK293T cells for 24 h, samples were harvested for Co-IP. The expression of relevant proteins was detected using Western blotting. (**E**) The QRD in hnRNP D inhibited IRF3-induced immune activation. Flag-tagged truncated hnRNP D, Myc-IRF3, IFN-β-Luc, and RL-TK were co-transfected into HEK293T cells. Twenty-four hours later, cells were lysed for Dual luciferase reporter gene assay. Relevant protein expression was detected by Western blotting. (**F**) HnRNP D inhibited the activation of PR8-induced IRF3 phosphorylation. After transfecting A549 cells with Flag-hnRNP D for 36 h, seeded with PR8 (MOI = 0.01) for 0, 8, and 12 h. Cells were harvested and lysed, and Western blotting was performed to detect the expression levels of relevant proteins. (**G**) HnRNP D reduced the levels of IRF3 protein that interacted with TBK1. HEK293T cells were co-transfected with Flag-TBK1 and a dose gradient of Myc-hnRNP D for 24 h. Then, cells were lysed and immunoprecipitated using anti-Flag antibody, and protein expression was detected by Western blotting. The data displayed are representative of three independent experiments. Bars represented the mean ± SD (*n* = 3). Values were analyzed by one-way ANOVA. Ns represented no significance, *P < 0.05, **P < 0.01.

Next, we attempted to identify the target nodes of within the innate immune signaling cascade targeted by hnRNP D. We co-transfected Myc-hnRNP D and Flag-tagged RIG-I, MAVS, TRAF3, TBK1, IKKε, or IRF3 into HEK293T cells and assessed IFN-β promoter activation. The results showed that hnRNP D was able to suppress the activation of IFN-β promoter induced by the above-mentioned key signaling molecules (Fig. 6A). Co-IP experiments confirmed that hnRNP D interacted with several components of this pathway, including RIG-I, MAVS, TRAF3, and IKKε and exhibited strong interaction with IRF3 during IAV infection (Fig. 6B). We then performed reverse immunoprecipitation using Flag-hnRNP D. Consistently, IRF3 interacted with hnRNP D both in virus-infected and non-infected cells (Fig. 7A). Cellular fractionation and microscopy observation revealed that while IRF3 coexisted in both the nucleus and cytoplasm, hnRNP D was predominantly localized within the nucleus with a lesser amount localized in the cytoplasm (Fig. 7B and C; Fig. S6A and B). Subsequently, we co-transfected HEK293T cells with truncated hnRNP D and IRF3, revealing that all truncations of hnRNP D could bind IRF3 (Fig. 7D), with the QRD being the key structural element responsible for inhibiting IRF3-induced activation of the IFN-β promoter (Fig. 7E). Since IRF3 activation relies on phosphorylation mediated by TBK1/IKKε (41), we next investigated this critical step. Notably, overexpression of hnRNP D significantly suppressed the phosphorylation levels of IRF3 during IAV infection (Fig. 7F). Further investigation into the relationship between hnRNP D and TBK1 revealed that while hnRNP D dose-dependently reduced the level of IRF3 that co-precipitated with TBK1 (Fig. 7G). In summary, these findings elucidated the mechanism by which hnRNP D weakened downstream interferon responses and thereby suppressed antiviral innate immunity by binding to IRF3 and preventing its phosphorylation by TBK1.

## DISCUSSION

HnRNPs play a crucial role in multiple stages of RNA metabolism, including RNA transcription, splicing, trafficking, and localization process. Their specific roles in regulating the replication processes of various viruses, including influenza viruses, are receiving increasing attention (20, 25, 42, 43). Among these, hnRNP D has emerged as a notable host factor, playing complex, situation-dependent roles in viral infections. As a suppressor, hnRNP D can inhibit virus replication via mechanisms such as repressing the splicing of human papillomavirus (HPV) 16 early mRNA (44), restraining poliovirus translation (45), and reducing hepatitis B virus (HBV) RNA stability (46). Conversely, hnRNP D also exerts positive regulatory effects on certain viruses. It significantly promotes the replication of West Nile virus (WNV) and other *Flavivirus* by facilitating viral genome synthesis (47–49), and promotes translation of the hepatitis C virus (HCV) protein (50). Notably, prior to this study, the impact of hnRNP D on IAV replication was entirely undefined. Here, we confirmed that hnRNP D p45 exists in interaction with all core components of the IAV vRNP (PB2, PB1, PA, and NP), and these interactions work together to inhibit viral polymerase activity and vRNP assembly. Paradoxically, in marked contrast to this antiviral effect, hnRNP D acts as a potent promoter of IAV replication in A549 cellular model. This contradictory phenomenon clearly refers to the presence of another antagonistic mechanism, which ultimately enables the virus to gain an advantage.

Several studies have revealed that host proteins may exhibit both antiviral and proviral effects on IAV. For instance, as an interferon-stimulated gene, IFIT2 can be utilized by IAV to promote the translation of viral mRNA, thereby facilitating viral replication (51). Tripartite motif 31 (TRIM31) exhibits dual regulatory mechanisms against IAV: it can both induce innate immune responses to suppress IAV replication and promote the stability of viral proteins to enhance IAV replication (40). In our study, we demonstrated through Vero E6 cells infection assays that hnRNP D suppresses PR8 replication in the absence of interferon, consistent with its function in inhibiting polymerase activity. This also validated that the proviral effect of hnRNP D in the A549 cellular model is closely related to innate immune regulation. The roles of hnRNPs in regulating innate antiviral immunity are complex and appear to be virus-specific. For example, hnRNP A2/B1 has been reported to enhance host recognition of DNA viruses, initiating and amplifying the production IFN-I (52); however, it can be redirected by the NSP1 protein of SARS-CoV-2 to attenuate the innate immune response (53). Similarly, hnRNP U has been confirmed to negatively regulate immunity by degrading TRAF3 mRNA during porcine epidemic diarrhea virus (PEDV) infection (54), yet another study demonstrated it enhances antiviral immune gene transcription during VSV infection (55). Only a few studies have specifically investigated the immunomodulatory functions of hnRNPs during IAV infection. Chicken hnRNPH2 was shown to inhibit the production of chIFN-β by disrupting the interaction between chMDA5 and MAVS during H5N6 and H9N2 infections (56), and hnRNP U interacts with the long non-coding RNA (lncRNA) to positively regulate IFN-β1 in the period of H1N1 infection (57). Our study filled this gap by identifying hnRNP D as a novel potent negative regulator of the innate immune response to IAV. We demonstrated that hnRNP D interacts with IRF3 and inhibits the binding of TBK1 to IRF3, thereby suppressing the phosphorylation and activation of IRF3. This disruption of a key kinase-substrate interaction represents a precise and effective strategy for viral immune evasion.

HnRNP D contains two distinct RRMs and a QRD (58). Our structural functional analysis revealed that both RRMs and QRD participate in inhibiting IAV polymerase activity. Strikingly, however, the intact hnRNP D p45 promotes viral replication in A549 cells primarily through its immune-suppressive function, with the QRD being the key domain responsible for suppressing IRF3-mediated immune activation. These findings offer a novel perspective on exploring the relationship between host proteins and viral infection: distinct domains within a single host protein can exert inconsistent regulatory effects on the viral life cycle. The final outcome, whether limiting or contributing to infection, likely depends on the balance between these specific functional domains and the cellular context.

IAVs have developed multiple approaches to hijack host factors, enabling them to evade the immune system. IPAN, a host lncRNA, has been proved to stabilize PB1 by interfering with RIG-I/TRIM25-mediated degradation (59). Yes-associated protein (YAP) and its analog protein, transcriptional coactivator with PDZ-binding motif (TAZ) are activated during IAV infection, thereby suppressing TLR3-induced immune responses (60). Furthermore, the viral PB1 protein promotes the degradation of MAVS by recruiting host proteins such as *BRCA1* (NBR1) (61) and TRIM25 (62). Similarly, we observed a transient upregulation of hnRNP D protein and mRNA levels during the early stages of viral infection (Fig. S7A and B). We hypothesize that IAV may actively exploit this early induction of hnRNP D to suppress the IFN response, leading to immune escape. Interestingly, in the later stages of infection, hnRNP D expression is downregulated. This may result from cell death caused by excessive viral invasion, or it may represent a host strategy to respond by restoring antiviral defense mechanisms. This dynamic regulation highlights the ongoing molecular tug-of-war between the virus and its host.

**Fig 8:**
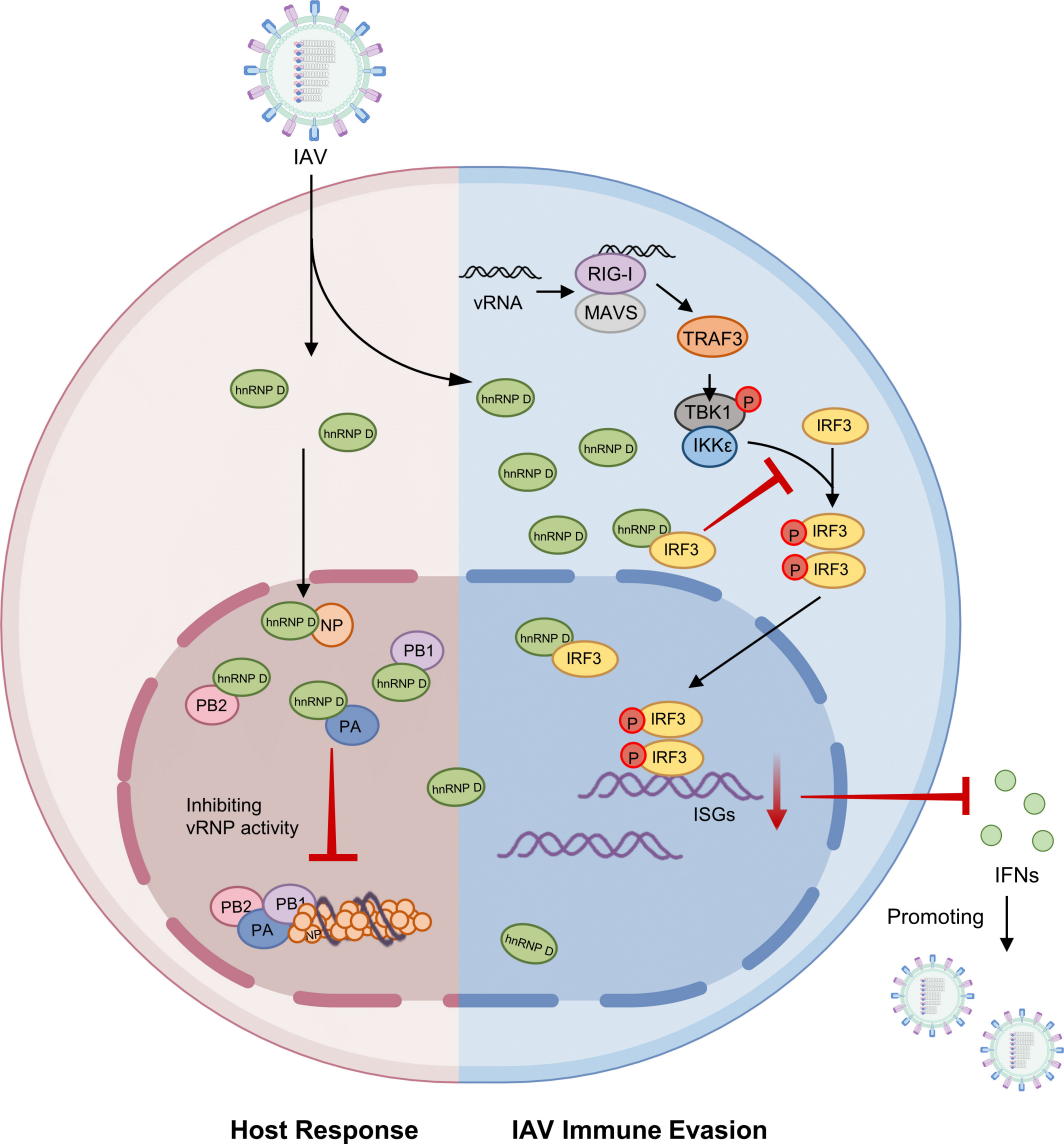
A model of the dual mechanism of hnRNP D in IAV replication. This model illustrated the opposing functions of hnRNP D. On one hand, hnRNP D acts as a negative regulator of the IAV polymerase, disrupting polymerase complex assembly and activity. On the other hand, viral infection increases the accumulation of hnRNP D, further enhancing its suppression of innate immunity and thereby significantly promoting viral replication. HnRNP D interacts with IRF3 and inhibited the binding between IRF3 and TBK1, blocking its phosphorylation and subsequent interferon production. The net outcome of enhanced viral replication in the typical A549 cellular model indicates the predominance of the immunosuppressive function.

In summary, we identified the host protein hnRNP D p45 as a positive regulator for IAV that operates through a dual regulatory mechanism. Although hnRNP D suppresses IAV polymerase activity, which is probably related to its regulation of the vRNP complex, it positively regulates viral replication in A549 cellular model, closely linked to its role in suppressing innate immune responses. We established that IAV is capable of suppressing TBK1 kinase activation of IRF3 by hijacking hnRNP D expression, thereby inhibiting IRF3 phosphorylation levels (Fig. 8). Overall, we have uncovered the effects of hnRNP D on regulating IAV replication, and understanding the intricate interplay between IAV and hnRNP D is crucial for developing effective strategies to combat influenza infections.

## MATERIALS AND METHODS

### Cells, viruses, and antibodies

Human embryonic kidney 293T (HEK293T) cells were maintained in Dulbecco’s modified Eagle’s medium (DMEM, Thermo Fisher Scientific, MA, USA) containing 10% fetal bovine serum (FBS, VivaCell, Shanghai, China) at 37°C with 5% CO_2_. Human lung adenocarcinoma A549 cells, Madin-Darby canine kidney (MDCK) cells and Vero E6 cells were maintained in DMEM containing 10% FBS (Thermo Fisher Scientific, MA, USA). *E. coli* BL21 (DE3) cells, A/Puerto Rico/8/1934 (PR8, H1N1), A/chicken/Zhejiang/A2013/2017 (A2013, H9N2), A/Anser fabalis/China/D322/2020 (D322, H3N1), Sendai virus (SeV), and GFP-expressing Vesicular Stomatitis Virus (VSV-GFP) were available in our laboratory. Anti-Flag mouse monoclonal antibody (F1804, Sigma Aldrich), anti-Myc rabbit polyclonal antibody (R1208-1, HUABIO), anti-GST mouse monoclonal antibody (sc-138, Santa Cruz Biotechnology), anti-GAPDH mouse monoclonal antibody (10021642, proteintech), anti-NP rabbit polyclonal antibody (GTX125989, GeneTex), anti-PB2 rabbit polyclonal antibody (GTX125926, GeneTex), anti-PB1 rabbit polyclonal antibody (GTX125923, GeneTex), anti-PA rabbit polyclonal antibody (GTX125932, GeneTex), anti-hnRNP D (Ab-83) rabbit polyclonal antibody (D151634, Sangon Biotech), anti-IRF3 rabbit polyclonal antibody (D220875, Sangon Biotech), anti-phospho-IRF3 (S386) recombinant rabbit monoclonal antibody (ET1608-22, HUABIO), horseradish peroxidase (HRP)-conjugated goat anti-mouse IgG antibody (5450-0011, KPL), HRP-conjugated goat anti-rabbit IgG antibody (5450-0010, KPL), DAPI staining solution (BL105A, Biosharp), Alexa Fluor 555-conjugated goat anti-rabbit IgG (bs-0295G-AF555, Bioss), and FITC-conjugated goat anti-mouse IgG (BL031A, Biosharp) were purchased from commercial sources.

### Construction of plasmids

The plasmids pCAGGS-Myc, pCAGGS-Flag, pCAGGS-Myc-PR8-PB2, pGEX-4T-1, pCAGGS-PB2, pcDNA-PB1, pcDNA-PA, pCAGGS-NP, phuPol I-Luc, and RL-TK were available in our laboratory. The hnRNP D gene was amplified from A549 cells and cloned into pCAGGS-Flag and pGEX-4T-1 vectors to generate expression plasmids for mammalian cells and GST-fusion protein, respectively. HnRNP D truncation mutants (RRMs, QRD, R + Q) were cloned from pCAGGS-Flag-hnRNP D and inserted into pCAGGS-Flag. PB2 truncation mutants (PB2-N, PB2-C) were cloned from pCAGGS-Myc-PR8-PB2 and inserted into pCAGGS-Myc. The Myc-tagged PB1, PA, and NP plasmids were cloned from infected A549 cell and inserted into pCAGGS-Myc. The RIG-I, MAVS, TRAF3, TBK1, IKKε, and IRF3 genes were amplified from A549 cells using specific primers, then cloned into pCAGGS-Flag or pCAGGS-Myc. The primers used were detailed in Table S1. PCR was performed using Phanta Max Super-Fidelity DNA polymerase (Vazyme, Nanjing, China), and plasmids constructs were generated using ClonExpress II One Step Cloning Kit (Vazyme, Nanjing, China) according to manufacturer’s instructions. All plasmid constructs were confirmed by sequencing. The constructed plasmids were transfected into cells using Jet Prime transfection reagent (Polyplus Transfection, Strasbourg, France) according to the manufacturer’s instructions.

### Mass spectrometry analysis

HEK293T cells expressing Myc-tagged PB2 or empty vector (control) were subjected to immunoprecipitation using an anti-Myc antibody. Purified protein complexes were analyzed by liquid chromatography-tandem mass spectrometry (LC-MS/MS) by GeneCreate Biological Engineering Company (Wuhan, China). All experiments were biologically repeated three times. When confidence ≥95%, unique peptides ≥1, and the unused score ≥1.3, the protein was identified as an interactor with viral proteins.

### Western blotting and co-immunoprecipitation

Cells were lysed with Cell lysis buffer for Western and IP (Beyotime, Shanghai, China) supplemented with Protease Inhibitor Cocktail (MedChemExpress, New Jersey, USA) or Phosphatase Inhibitor Cocktail II (MedChemExpress, New Jersey, USA). For immunoprecipitation, the cell lysate was added with 100 U/mL RNase inhibitor (Beyotime, Shanghai, China) or RNase A (Beyotime, Shanghai, China) incubated for 1 h at 4°C, followed by incubation with 5 μg of anti-Flag mouse monoclonal antibody for 4 h, then with protein A/G-agarose (sc-2003, Santa Cruz Biotechnology, USA) for 12 h at 4°C. Immunoprecipitated proteins were washed for five times and dissolved in Cell lysis buffer for Western and IP. For Western blotting, added 4× SDS PAGE Loading Buffer (Solarbio, Beijing, China) to the sample and heated at 95°C for 10 min. Proteins were separated by SDS-PAGE and transferred to 0.45 μm polyvinylidene fluoride (PVDF) membranes (Merck Millipore, MA, USA). The membranes were blocked with 5% skimmed milk in PBST and then incubated with specific antibodies and HRP-conjugated antibodies. Signals were detected using ECL buffer (2×) (Vazyme, Nanjing, China) and quantified using Image J software (NIH, USA).

### GST pull-down

GST or GST-hnRNP D fusion protein was expressed in *E. coli* BL21 (DE3) cells at 16°C for 16 h, and the supernatant was obtained by sonication. Then 1 mL supernatant was mixed with 60 μL Glutathione MagBeads (L00895, GenScript) for 4 h at 4°C. Beads bound to GST proteins were incubated with lysates from HEK293T cells expressing Myc-tagged PB2, PB1, PA, or NP for 4 h at 4°C. After five times washes, the bound proteins were analyzed by Western blotting.

### Overexpression of host proteins, viral infection and titration

The A549 cells were grown to over 80% confluence in 12-well plates and transfected with 0.8 μg of Flag-hnRNP D for 36 h. Then, the cells were infected with IAVs at the indicated MOI for 12 h. Cell lysate samples were used to detect the content of viral proteins by Western blotting. The MDCK cells were grown to over 80% confluence in 96-well plates and were then inoculated with the collected supernatant from infected cell cultures. After 72 h, detection was performed using anti-NP antibodies via immunofluorescence staining. The presence of fluorescence was considered positive. The TCID_50_ was determined by the Reed-Muench method.

### Gene silencing using siRNA

Human hnRNP D small interfering RNA (siRNA, sihnRNP D) and negative-control siRNA (siNC) listed in Table S2 were synthesized by GENEray (Shanghai, China). A549 cells were transfected with sihnRNP D or siNC for 36 h and then infected with IAVs at the indicated MOI for 12 hours before being harvested for Western blotting.

### Immunofluorescence staining and confocal microscopy

HEK293T cells or A549 cells at 60%–80% confluency on a confocal dish were co-transfected with specified plasmids for 24 h or were transfected with plasmids then followed by infecting with PR8 (MOI = 1) for 8 h. Cells were fixed with 4% paraformaldehyde for 20 min at room temperature and then washed with PBS for three times. Cells were permeabilized with 0.1% Triton X-100 in PBS for 10 mins, washed, and blocked with 1% BSA in PBS for 1 h at 37°C, followed by incubation with the corresponding antibodies for 2 h at 37°C. After being washed with PBS, the cells were incubated with corresponding fluorescent antibodies for 1 h at 37°C. After three times washes, the nuclei were stained with DAPI for 10 min. The images were acquired by confocal laser scanning microscope.

### RNA isolation and quantitative real-time PCR

Total RNA from infected or transfected cells was extracted by RNA isolater Total RNA Extraction Reagent (Vazyme, Nanjing, China) according to the manufacturer’s instructions and subsequently transcribed into cDNA using Hifair III 1st Strand cDNA Synthesis SuperMix for qPCR (Yeasen, Shanghai, China). Quantitative real-time PCR was performed using the Hieff UNICON qPCR SYBR Green Master Mix (Yeasen, Shanghai, China). Fold change of RNA levels normalized to GAPDH was calculated by the 2-ΔΔCt method. All primers were listed in Table S3.

### Dual luciferase reporter gene assay

HEK293T cells were co-transfected with *Firefly* luciferase reporter plasmids (IFN-β-Luc or ISRE-Luc), a control *Renilla* luciferase plasmid (RL-TK), and expression plasmids or siRNA as indicated. At 24 h post-transfection, cells were infected with SeV (MOI = 0.1) or transfecting with 3 μg Poly (I:C) (Invitrogen, CA, USA) for 12 h. Cells lysed at room temperature, and the supernatant was equilibrated to room temperature and added to a 96-well white plate and then was detected by Dual Luciferase Reporter Gene Assay Kit (Beyotime, Shanghai, China). *Firefly* luciferase detection reagent and *Renilla* luciferase detection reagent were added sequentially, and the relative light unit (RLU) was read. The final result was calculated by dividing the *Firefly* luciferase RLU by the *Renilla* luciferase RLU value.

### Statistical analysis

The study data were presented as the mean ± SD at least three independent experiments. Statistical significance was analyzed using Student’s *t*-test (for two groups) or one-way ANOVA (for multiple groups) in GraphPad Prism 9.0.0 software (GraphPad Software, San Diego, CA, USA). The *P*-values in the figures are defined as follows: ns represented no significance, *P < 0.05, **P < 0.01, ***P < 0.001, ****P < 0.0001.

## Data Availability

The nucleotide sequences of hnRNP D p45 and its variants are available through NCBI (p37: NM_001003810.2, p40: NM_002138.4, p42: NM_031369.3). All other data are included in the article and supplemental material.

## SUPPLEMENTAL MATERIAL

Supplemental material jvi.00257-26-s0001.docx Fig. S1 to S7; Tables S1 to S3.

ASM does not own the copyrights to Supplemental Material that may be linked to, or accessed through, an article. The authors have granted ASM a non-exclusive, world-wide license to publish the Supplemental Material files. Please contact the corresponding author directly for reuse.
