# Supplementary material for: Cellular hnRNP D promotes influenza A virus replication by inhibiting TBK1-IRF3-mediated innate immune response
Source: J Virol. 2026 May 13;100(6):e00257-26. doi: 10.1128/jvi.00257-26 (PMC13288638; doi:10.1128/jvi.00257-26)
Supplement: Supplemental material — Fig. S1 to S7; Tables S1 to S3. [file jvi.00257-26-s0001.docx]

**SUPPLEMENTAL MATERIAL**

**Cellular hnRNP D promotes influenza A virus replication by inhibiting TBK1-IRF3-mediated innate immune response**

Chenchen Xu ^a^, Yunling Peng ^a^, Shuhui Liu ^a^, Ran Xie ^a^, Duanchenxi Feng ^a^, Zhenwei Bi ^a, b^, Liping Yan ^a, #^

^a^ MOE Joint International Research Laboratory of Animal Health and Food Safety, Jiangsu Detection Center of Terrestrial Wildlife Disease, Institute of Immunology and College of Veterinary Medicine, Nanjing Agricultural University, Nanjing, Jiangsu 210095, China

^b^ Institute of Veterinary Medicine, Jiangsu Academy of Agricultural Sciences, Key Laboratory of Veterinary Biological Engineering and Technology, Ministry of Agriculture and Rural Affairs, Nanjing, Jiangsu, 210014, China

**Running Head**: HnRNP D promotes influenza A virus replication.

^#^Address correspondence to Liping Yan, [yanliping@njau.edu.cn](mailto:yanliping@njau.edu.cn).


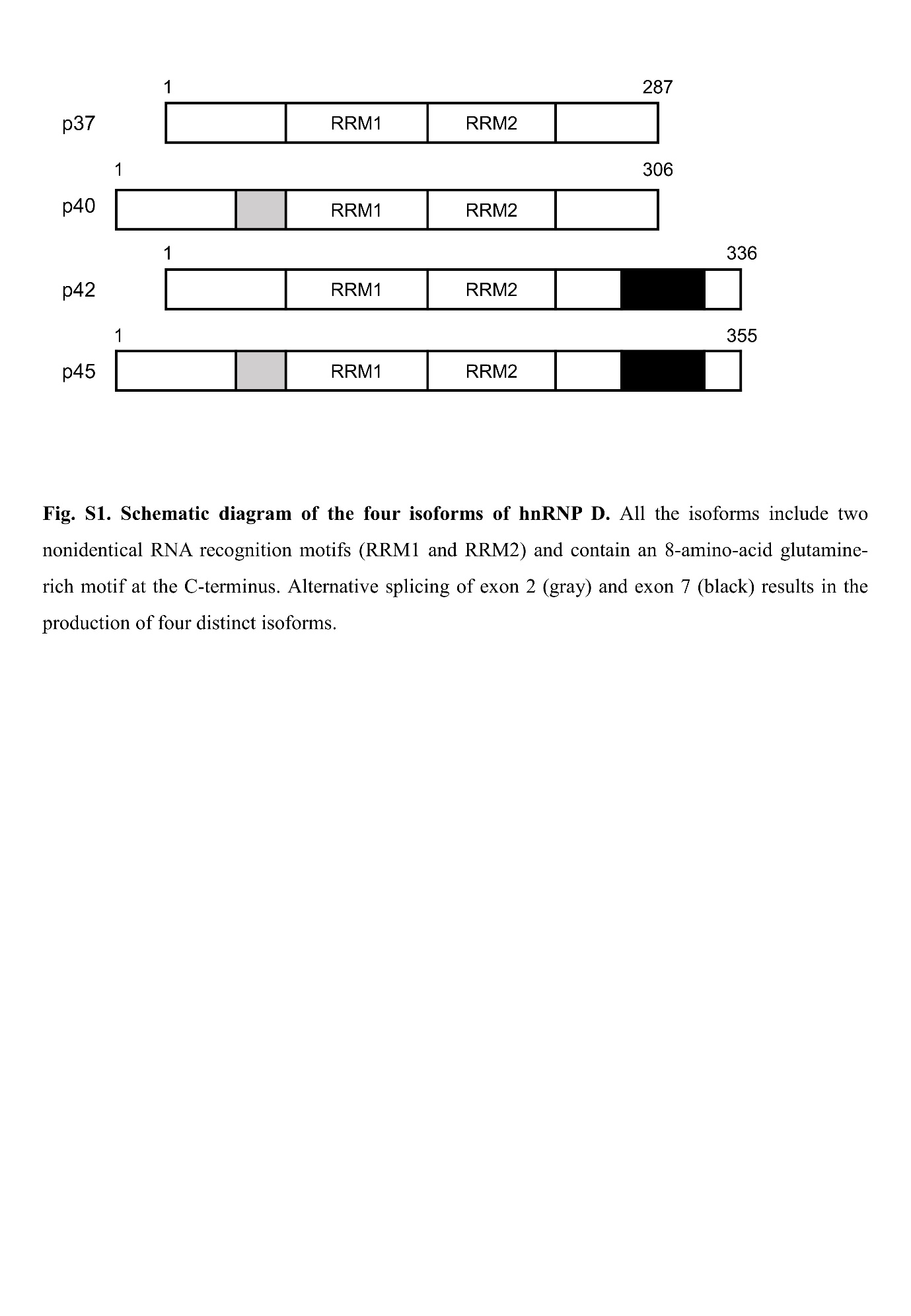


**Fig S1. Schematic diagram of the four isoforms of hnRNP D.** All the isoforms include two nonidentical RNA recognition motifs (RRM1 and RRM2) and contain an 8-amino-acid glutamine-rich motif at the C-terminus. Alternative splicing of exon 2 (gray) and exon 7 (black) results in the production of four distinct isoforms.


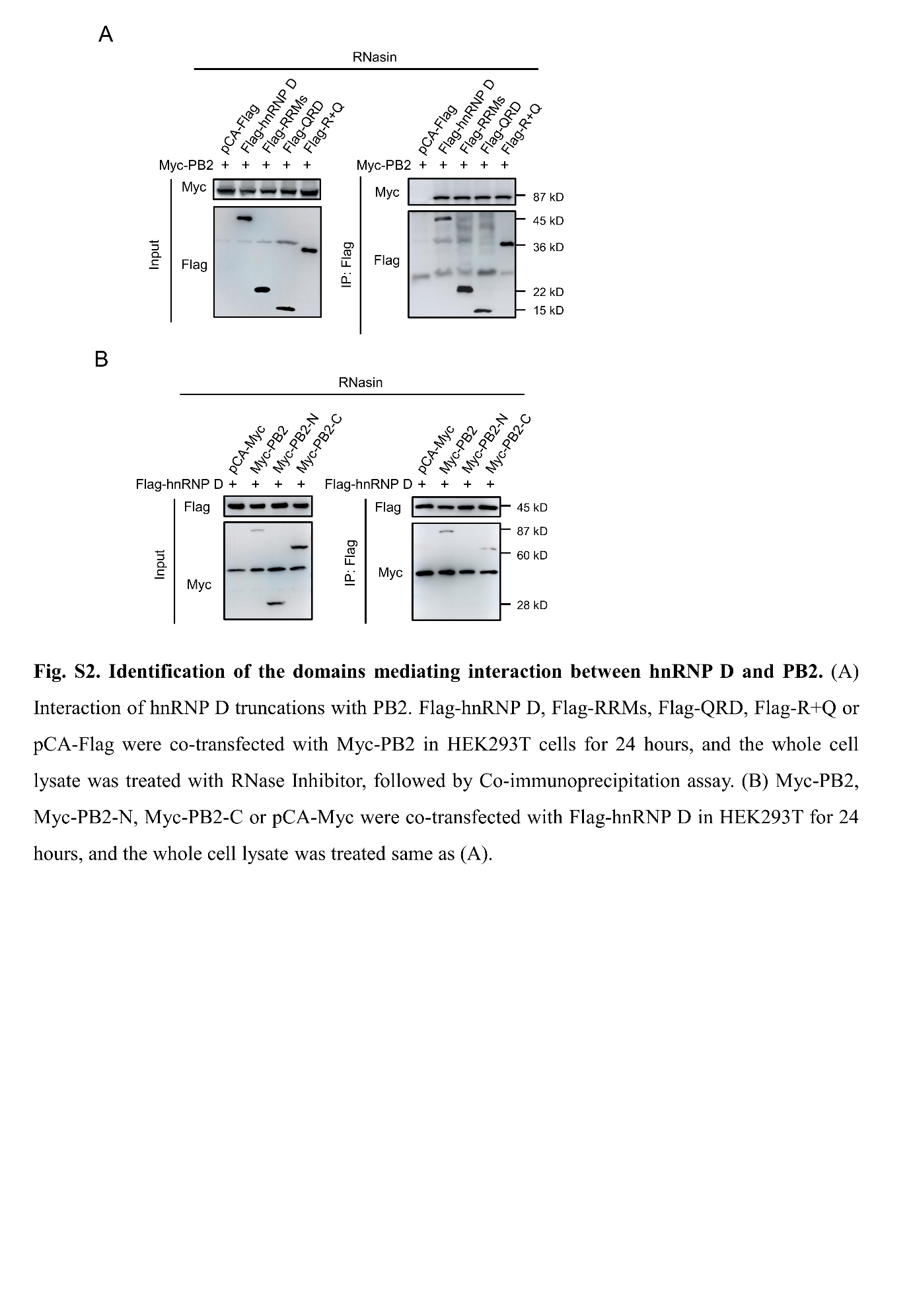


**Fig S2. Identification of the domains mediating interaction between hnRNP D and PB2.** (A) Interaction of hnRNP D truncations with PB2. Flag-hnRNP D, Flag-RRMs, Flag-QRD, Flag-R+Q or pCA-Flag were co-transfected with Myc-PB2 in HEK293T cells for 24 hours, and the whole cell lysate was treated with RNase Inhibitor, followed by Co-immunoprecipitation assay. (B) Myc-PB2, Myc-PB2-N, Myc-PB2-C or pCA-Myc were co-transfected with Flag-hnRNP D in HEK293T for 24 hours, and the whole cell lysate was treated same as (A).


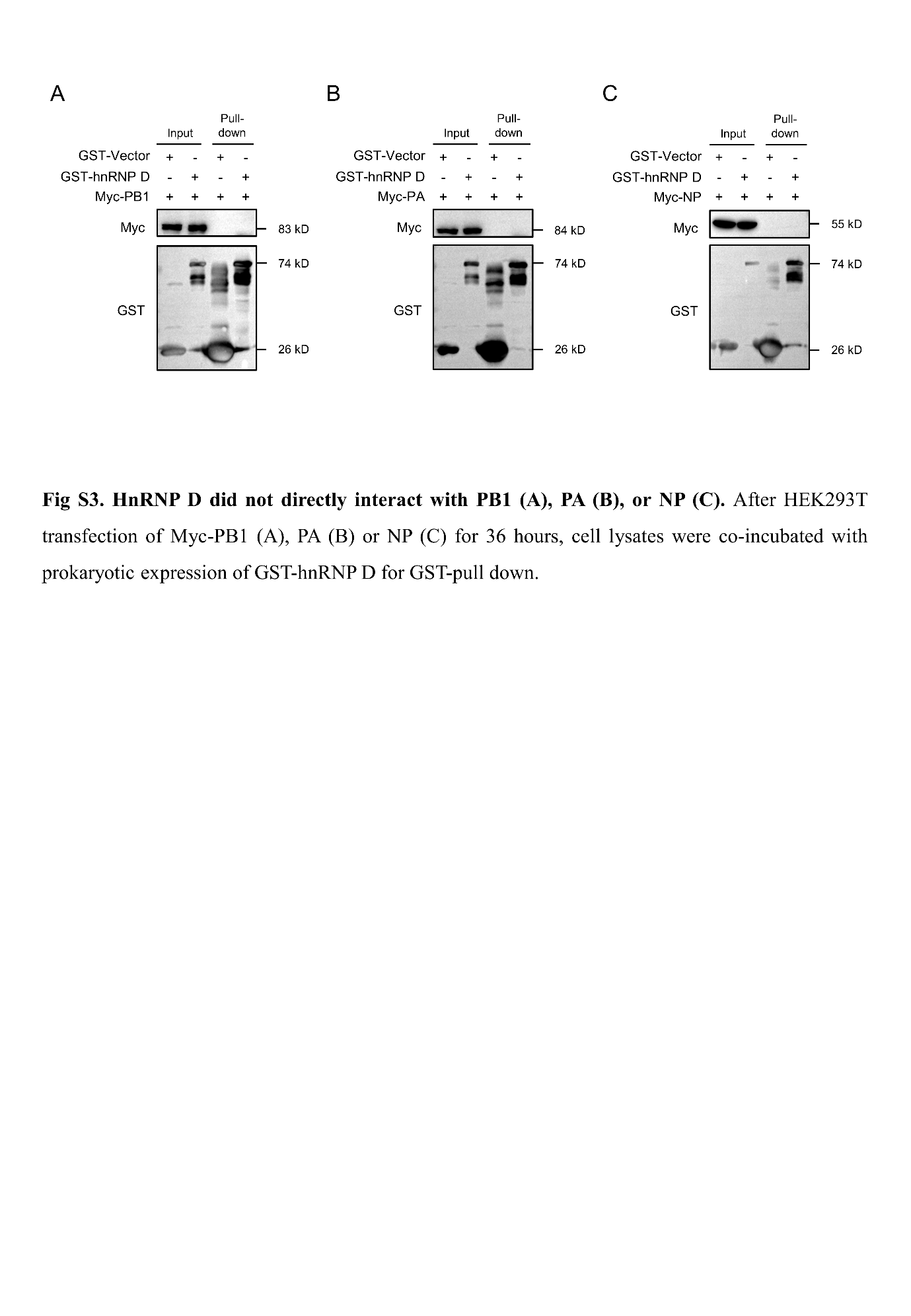


**Fig S3. HnRNP D does not directly interact with PB1 (A), PA (B), or NP (C).** After HEK293T transfection of Myc-PB1 (A), PA (B) or NP (C) for 36 hours, cell lysates were co-incubated with prokaryotic expression of GST-hnRNP D for GST-pull down.


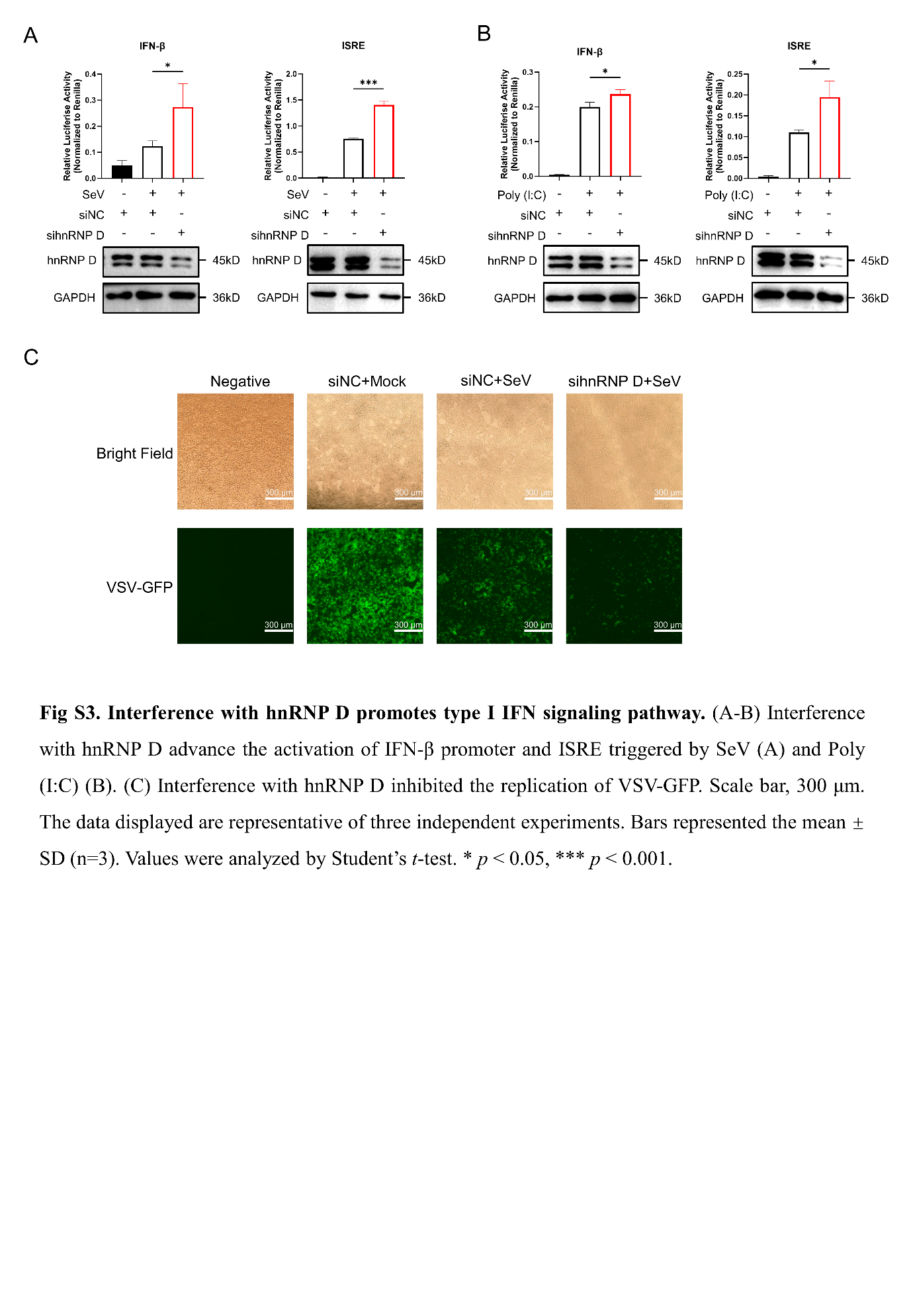


**Fig S4. Interference with hnRNP D promotes type I IFN signaling pathway.** (A-B) Interference with hnRNP D advance the activation of IFN-β promoter and ISRE triggered by SeV (A) and Poly (I:C) (B). (C) Interference with hnRNP D inhibited the replication of VSV-GFP. Scale bar, 300 μm. The data displayed are representative of three independent experiments. Bars represented the mean ± SD (n=3). Values were analyzed by Student’s *t*-test. * *p* < 0.05, *** *p* < 0.001.


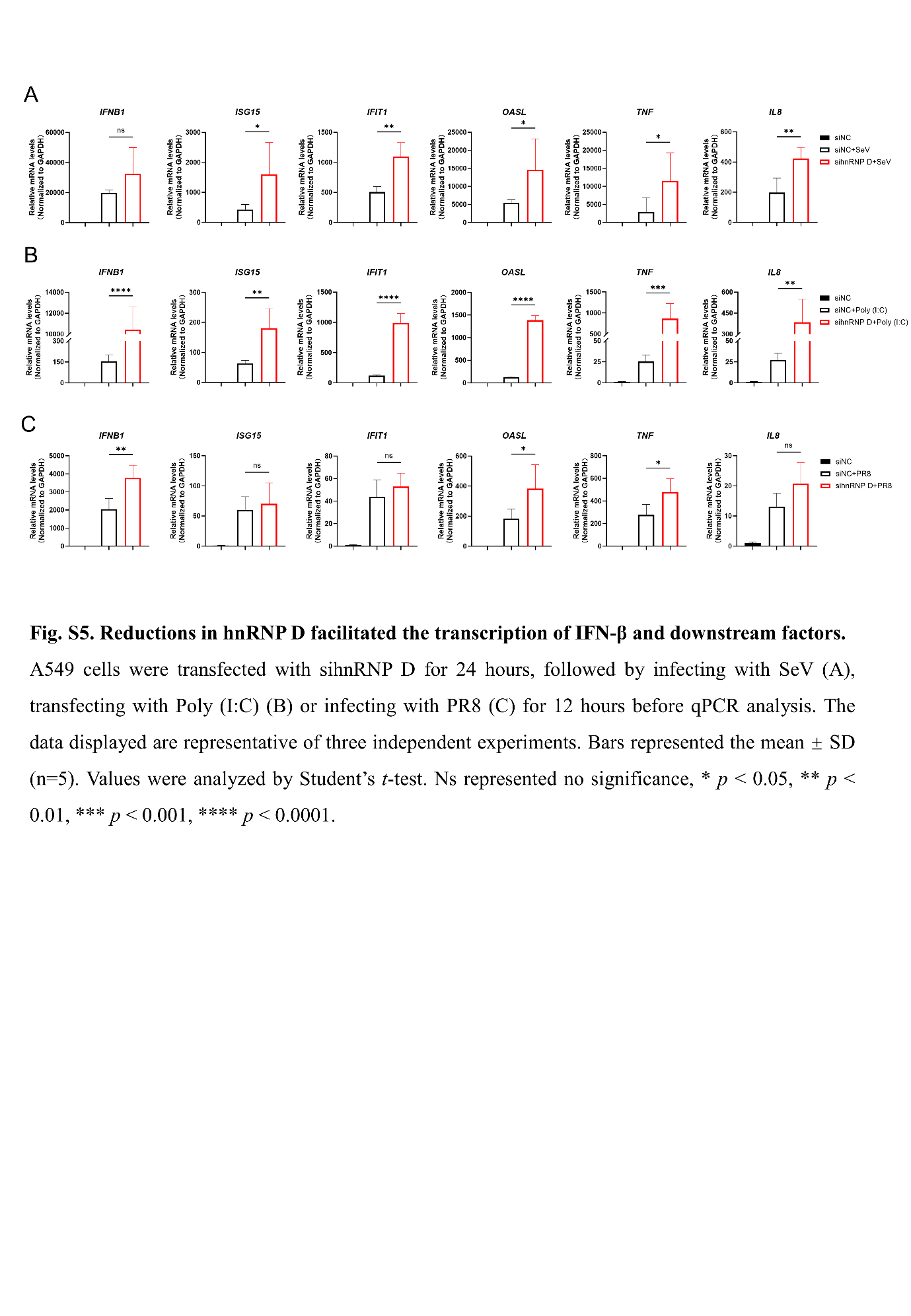


**Fig S5. Reductions in hnRNP D facilitates the transcription of IFN-β and downstream factors.** A549 cells were transfected with sihnRNP D for 24 hours, followed by infecting with SeV (A), transfecting with Poly (I:C) (B) or infecting with PR8 (C) for 12 hours before qPCR analysis. The data displayed are representative of three independent experiments. Bars represented the mean ± SD (n=5). Values were analyzed by Student’s *t*-test. Ns represented no significance, * *p* < 0.05, ** *p* < 0.01, *** *p* < 0.001, **** *p* < 0.0001.


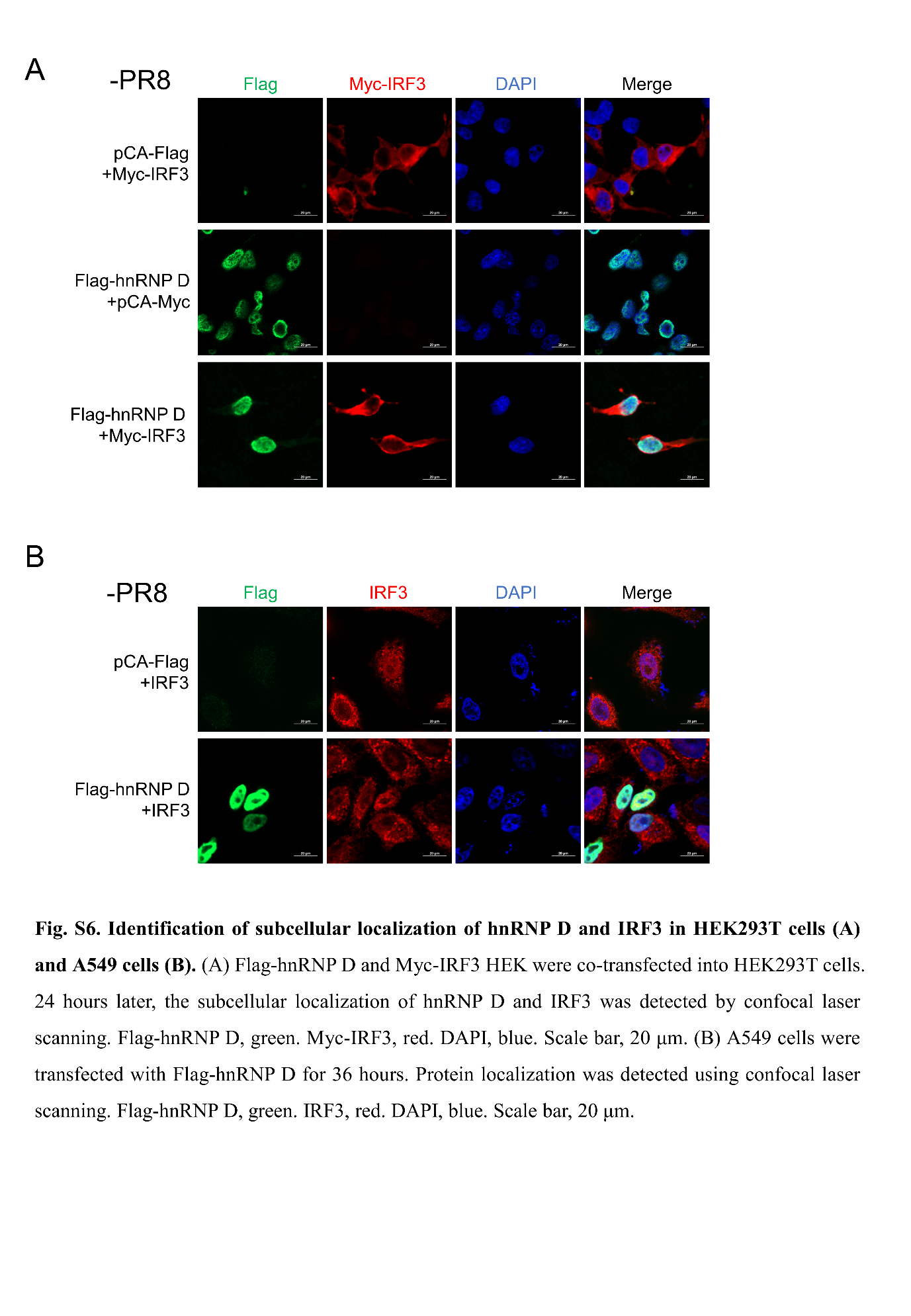


**Fig S6. Identification of subcellular localization of hnRNP D and IRF3 in HEK293T cells (A) and A549 cells (B).** (A) Flag-hnRNP D and Myc-IRF3 were co-transfected into HEK293T cells. 24 hours later, the subcellular localization of hnRNP D and IRF3 was detected by confocal laser scanning. Flag-hnRNP D, green. Myc-IRF3, red. DAPI, blue. Scale bar, 20 μm. (B) A549 cells were transfected with Flag-hnRNP D for 36 hours. Protein localization was detected using confocal laser scanning. Flag-hnRNP D, green. IRF3, red. DAPI, blue. Scale bar, 20 μm.


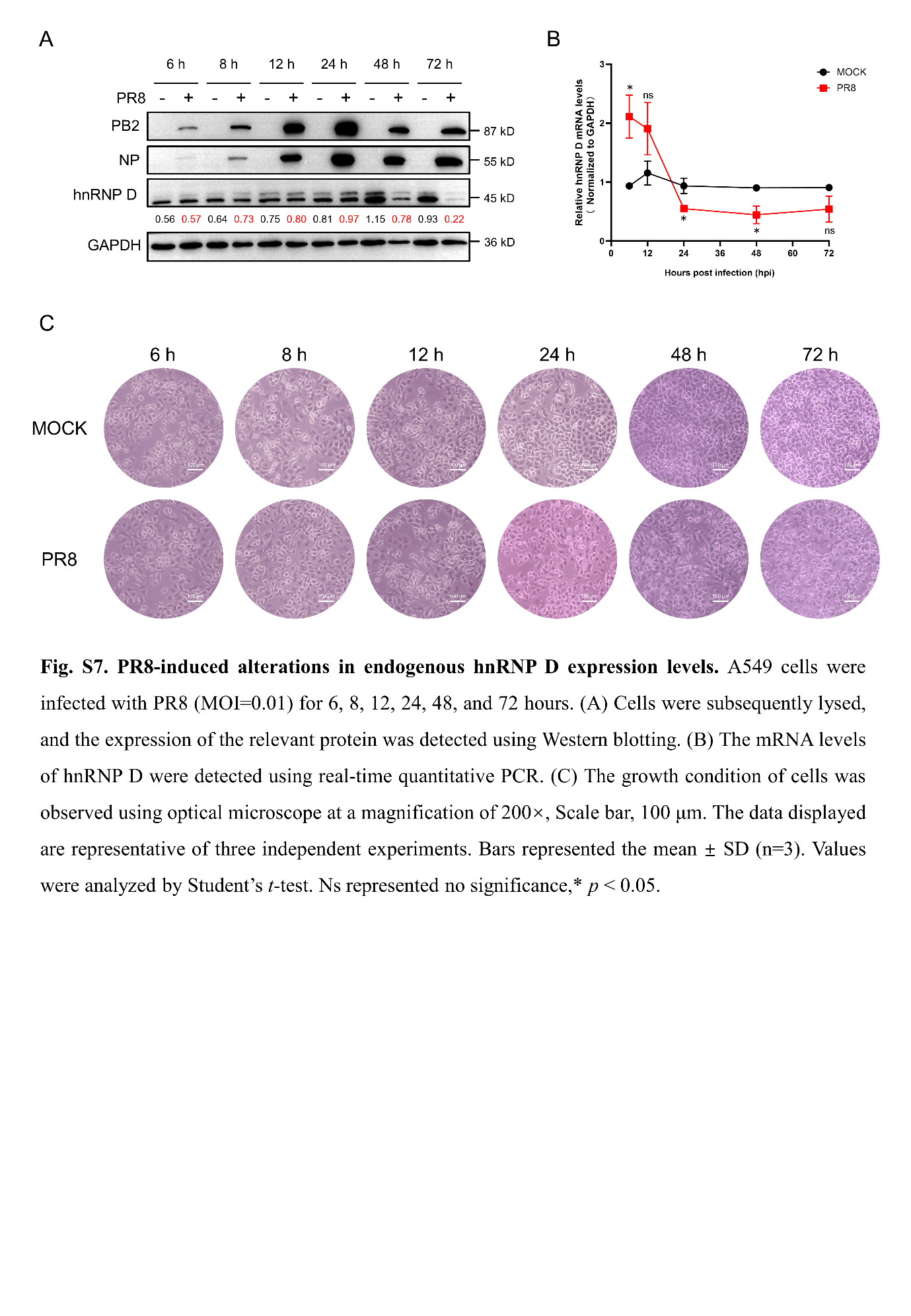


**Fig S7. PR8-induced alterations in endogenous hnRNP D expression levels.** A549 cells were infected with PR8 (MOI=0.01) for 6, 8, 12, 24, 48, and 72 hours. (A) Cells were subsequently lysed, and the expression of the relevant protein was detected using Western blotting. (B) The mRNA levels of hnRNP D were detected using real-time quantitative PCR. (C) The growth condition of cells was observed using optical microscope at a magnification of 200×, Scale bar, 100 μm. The data displayed are representative of three independent experiments. Bars represented the mean ± SD (n=3). Values were analyzed by Student’s *t*-test. Ns represented no significance, * *p* < 0.05.

**Table S1. Primers used in constructing plasmids.**

| Primers | Sequences (5’-3’) |
| --- | --- |
| Flag-huhnRNP D-F | *cacgacgatgacaa*ATGTCGGAGGAGCAGTTC |
| Flag-huhnRNP D-R | *agggaaaaagatctg*TTAGTATGGTTTGTAGCTATTTTG |
| GST-huhnRNP D-F | *ccgcgtggatccccggaattc*ATGTCGGAGGAGCAGTTC |
| GST-huhnRNP D-R | *gtcacgatgcggccgctcgag*TTAGTATGGTTTGTAGCTATTTTGATG |
| Flag-RRMs-F | *cacgacgatgacaag*ATGTTTATAGGAGGCCTTAGCT |
| Flag-RRMs-R | *agggaaaaagatctg*TTAGGCTACTTTTATTTCACAT |
| Flag-QRD-F | *cacgacgatgacaag*ATGTCGAAGGAACAATATCAGC |
| Flag-QRD-R | *agggaaaaagatctg*TTAGTATGGTTTGTAGCTATTT |
| Myc-PB2-N-F | *ctcatctctgaagag*ATGGAAAGAATAAAAGAACTAAGAA |
| Myc-PB2-N-R | *aaaaagatctgctag*CTATCCTGGAGTATACATCTGTTCCC |
| Myc-PB2-C-F | *ctcatctctgaagag*ATGGGGGAAGTGAGGAATGATGAT |
| Myc-PB2-C-R | *aaaaagatctgctag*CTAATTGATGGCCATCCGAATTCTT |
| Myc-PB1-F | *ctcatctctgaagag*ATGGATGTCAATCCGACCTT |
| Myc-PB1-R | *aaaaagatctgctag*CTATTTTTGCCGTCTGAGCTC |
| Myc-PA-F | *ctcatctctgaagag*ATGGAAGATTTTGTGCGACAATG |
| Myc-PA-R | *aaaaagatctgctag*CTAACTCAATGCATGTGTAAGGAAGG |
| Myc-NP-F | *ctcatctctgaagag*ATGGCGTCCCAAGGCACCAAAC |
| Myc-NP-R | *aaaaagatctgctag*TTAATTGTCGTACTCCTCTGCAT |
| Flag-RIG-Ⅰ-F | *cacgacgatgacaag*ATGACCACCGAGCAGCGA |
| Flag-RIG-Ⅰ-R | *agggaaaaagatctg*TCATTTGGACATTTCTGCTGG |
| Flag-MAVS-F | *cacgacgatgacaag*ATGCCGTTTGCTGAAGACAAG |
| Flag-MAVS-R | *agggaaaaagatctg*CTAGTGCAGACGCCGCCGGTA |
| Flag-TRAF3-F | *cacgacgatgacaag*ATGGAGTCGAGTAAAAAGATGGAC |
| Flag-TRAF3-R | *agggaaaaagatctg*TCAGGGATCGGGCAGATCCGAA |
| Flag-TBK1-F | *cacgacgatgacaag*ATGCAGAGCACTTCTAATCATCTG |
| Flag-TBK1-R | *agggaaaaagatctg*CTAAAGACAGTCAACGTTGCG |
| Flag-IKKε-F | *cacgacgatgacaag*ATGCAGAGCACAGCCAATT |
| Flag-IKKε-R | *agggaaaaagatctg*TCAGACATCAGGAGGTGCT |
| Flag-IRF3-F | *cacgacgatgacaag*ATGGGAACCCCAAAGCCA |
| Flag-IRF3-R | *agggaaaaagatctg*TCAGCTCTCCCCAGGGC |
| Myc-IRF3-F | *ctcatctctgaagag*ATGGGAACCCCAAAGCCA |
| Myc-IRF3-R | *aaaaagatctgctag*TCAGCTCTCCCCAGGGC |

Note: The homologous arms of the plasmids are indicated in italicized portions.

**Table S2. Specific small interfering RNA sequences of homo hnRNP D.**

| siRNA | Sequences (5’-3’) |
| --- | --- |
| sihnRNP D-sense | CAAUGUUGGUCUUAGUAAATT |
| sihnRNP D-antisense | UUUACUAAGACCAACAUUGTT |
| siNC-sense | UUCUCCGAACGUGUCACGUTT |
| siNC-antisense | ACGUGACACGUUCGGAGAATT |

**Table S3. Primers used in quantitative real-time PCR.**

| Primers | Sequences (5’-3’) |
| --- | --- |
| qIFNB1-F | GCTTGGATTCCTACAAAGAAGCA |
| qIFNB1-R | ATAGATGGTCAATGCGGCGTC |
| qISG15-F | CCTTCCAGCAGCGTCTGG |
| qISG15-R | GGTTCGTCGCATTTGTCC |
| qIFIT1-F | TCATCAGGTCAAGGATAGTC |
| qIFIT1-R | CACACTGTATTTGGTGTCTAGG |
| qOASL-F | CTGATGCAGGAACTGTATAGCAC |
| qOASL-R | CACAGCGTCTAGCACCTCTT |
| qTNF-F | ACTTTGGAGTGATCGGCC |
| qTNF -R | GCTTGAGGGTTTGCTACAAC |
| qIL8-F | GGTGCAGTTTTGCCAAGGAG |
| qIL8-R | TTCCTTGGGGTCCAGACAGA |
| qhnRNP D-F | GGAGGGGGCGAAGATTG |
| qhnRNP D-R | TGTCGTGGGGAGGAGTT |
| qGAPDH-F | GTCTCCTCTGACTTCAACAGCG |
| qGAPDH-R | ACCACCCTGTTGCTGTAGCCAA |
